# High-Level Ab Initio Predictions of Thermochemical Properties of Organosilicon Species: Critical Evaluation of Experimental Data and a Reliable Benchmark Database for Extending Group Additivity Approaches

**DOI:** 10.1021/acs.jpca.1c09980

**Published:** 2022-03-07

**Authors:** Hannu T. Vuori, J. Mikko Rautiainen, Erkki T. Kolehmainen, Heikki M. Tuononen

**Affiliations:** Department of Chemistry, Nanoscience Centre, University of Jyväskylä, P.O. Box 35, Jyväskylä FI-40014, Finland

## Abstract

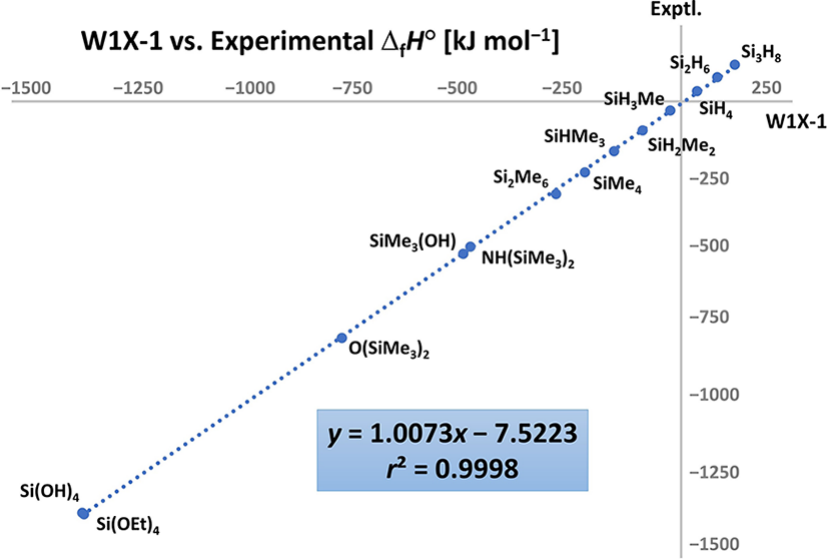

A high-level composite quantum chemical method, W1X-1, is used herein to calculate the gas-phase standard enthalpy of formation, entropy, and heat capacity of 159 organosilicon compounds. The results set a new benchmark in the field that allows, for the first time, an in-depth assessment of existing experimental data on standard enthalpies of formation, enabling the identification of important trends and possible outliers. The calculated thermochemical data are used to determine Benson group additivity contributions for 60 Benson groups and group pairs involving silicon. These values allow fast and accurate estimation of thermochemical parameters of organosilicon compounds of varying complexity, and the data acquired are used to assess the reliability of experimental work of Voronkov et al. that has been repeatedly criticized by Becerra and Walsh. Recent results from other computational investigations in the field are also carefully discussed through the prism of reported advancements.

## Introduction

A central concept in thermochemistry is the standard enthalpy of formation of a compound, Δ_f_*H*°, the enthalpy change during the formation of one mole of a particular substance from its elements with all constituents in their standard states.^1^ Standard enthalpies of formation are typically determined from experimentally measured standard enthalpies of combustion and by applying Hess’s law of constant heat summation. This also elucidates the centrality of Δ_f_*H*° in thermochemistry as the enthalpy change of any reaction, Δ_r_*H*°, can be calculated (eq 1) by taking the difference in the sum of standard enthalpies of formation of the products (i) and that of the reactants (j), with each value multiplied by its stoichiometric coefficient ν_n_:
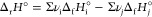
1

Standard enthalpies of formation are often determined using calorimetry.^2^ The approach is straightforward for many organic compounds and requires a measurement of the enthalpy of combustion of the compound in question along with literature data for the combustion products, such as CO_2_, H_2_O, and NO_*x*_. Although simple in principle, combustion calorimetry is much more laborious in practice. For example, the required measurements of weight and temperature must be conducted to high precision and all undesirable side reactions, such as incomplete combustion or oxidation of the crucible, appropriately accounted for. Furthermore, reactions involving very small heat changes are challenging for combustion calorimetry, as is also true for compounds that are volatile, highly reactive, or slowly burning.

During the past two decades, high-accuracy quantum chemical methods have gained ground as important alternatives to accurately determine standard enthalpies of formation.^3^ The Weizmann-1 (W1) method was the first widely applicable protocol to reach chemical accuracy (a mean absolute deviation, MAD, less than 4 kJ mol^–1^) for second- and third-row compounds,^4^ while more advanced methods, such as W4,^5^ FPD,^6^ and HEAT-QP,^7^ are nowadays able to predict standard enthalpies of formation even at sub-kJ mol^–1^ precision. The caveat with using the most accurate methods is that they can only be applied to molecules with less than 10 non-hydrogen atoms, and even W1 and its variants can only effectively handle systems up to 20 heavy atoms.^3^

A practical alternative for obtaining thermodynamic data on larger molecules without conducting experimental measurements is to use group additivity approaches. These are based on the century-old empirical notion that the properties of molecules can be accurately estimated by dividing them into groups whose contributions to physical properties remain nearly constant from one system to another.^8^ The scheme originally proposed by Benson and Buss,^9^ later extended to liquid and solid phases by Domalski and Hearing,^10,11^ has become one of the most successful realizations of this kind. While it might seem archaic in the era of high-performance computing, the approach is very powerful when data are needed on a large group of molecules, so that quantum chemical methods, be they of any kind, would be too time-consuming. This is especially true in combustion chemistry and automated reaction mechanism generation in particular.^12^

The accuracy of group additivity approaches naturally depends on two factors: how well the additivity approximation holds and how accurate the data used to determine the group contributions are. Even though the additivity of properties is not strictly fulfilled beyond atomic and molecular masses, experience accumulated over the past 50+ years has shown that Benson’s methodology is able to achieve chemical accuracy for many organic systems, that is, molecules composed of atoms H, B, C, N, O, F, P, S, Cl, Br, and I.^13,14^ Thus, important keys to the success of group additivity approaches are the treatment of molecules that are problematic for additivity, such as strained or sterically congested systems, and ensuring that the reference thermochemical data used to determine the group contributions are of the highest quality.

As noted by Benson,^15^ multiple inconsistencies in the experimental standard enthalpies of formation of organosilicon compounds prevented the determination of an internally consistent set of additivity contributions for silicon-based groups. It is now well established that many of the pre-1970 calorimetric experiments on silicon compounds were in error due to incomplete combustion,^16^ and the associated values have largely been removed from thermochemical compilations. Unfortunately, what remained in the data libraries became less than comprehensive and the situation has not improved significantly over the years. As repeatedly discussed by Walsh and Becerra in their excellent reviews on the thermochemistry of organosilicon compounds,^17−19^ the science of calorimetry has become almost extinct, and only a very few experimental values have been published for silicon-based species during the past 25 years. Furthermore, even the ones that have been published, such as the very comprehensive works of Voronkov et al.,^20−25^ have been questioned to be affected by systematic error(s) due to their incompatibility with other literature values that are often associated with high uncertainties themselves.

The aim of the current contribution is threefold. First, we use the high-level W1X-1 composite method^26^ to calculate the standard enthalpies of formation of 159 organosilicon species. To the best of our knowledge, this is the first comprehensive effort to establish a high-accuracy ab initio thermochemical benchmark database for organosilicon compounds. The calculated values are compared with experimental data, where available, allowing us to assess their accuracy and to pinpoint outliers and other inconsistencies. Of special interest are the results published by Voronkov et al.,^20−25^ in which case our aim is to determine whether the practice of flagging their data in thermochemical reviews is entirely justified. Second, we compare our results to the earlier CCSD(T)/CBS benchmark values of Feller and Dixon,^27^ as well as to the recent computational works of Burcat and Goos^28^ and Janbazi et al.^29,30^ The data reported by Janbazi et al. are found to be partially inconsistent with the other results, which is not only problematic by itself but also because the G4 enthalpies have been used as reference data in establishing group additivity contributions. Third, after carefully evaluating the reliability of our thermochemical data, we use the W1X-1 results to derive group additivity contributions for the standard gas-phase enthalpy of formation, Δ_f_*H*_298K_^°^, entropy, *S*_298K_^°^, and heat capacity, *C*_*p*_, for 60 Benson groups and group pairs involving silicon. We show that the group contributions form internally consistent sets and compare them to those reported by Walsh and Becerra^18^ and Janbazi et al.^29,30^ We also use our group contributions to determine the standard enthalpies of formation for several organosilicon species examined by Voronkov et al.^20−25^ that could not be calculated directly with the W1X-1 method simply due to molecular size. This allows a thorough assessment of the experimental data published by Voronkov et al. for possible systematic errors.

## Computational Details

**Chart 1:**
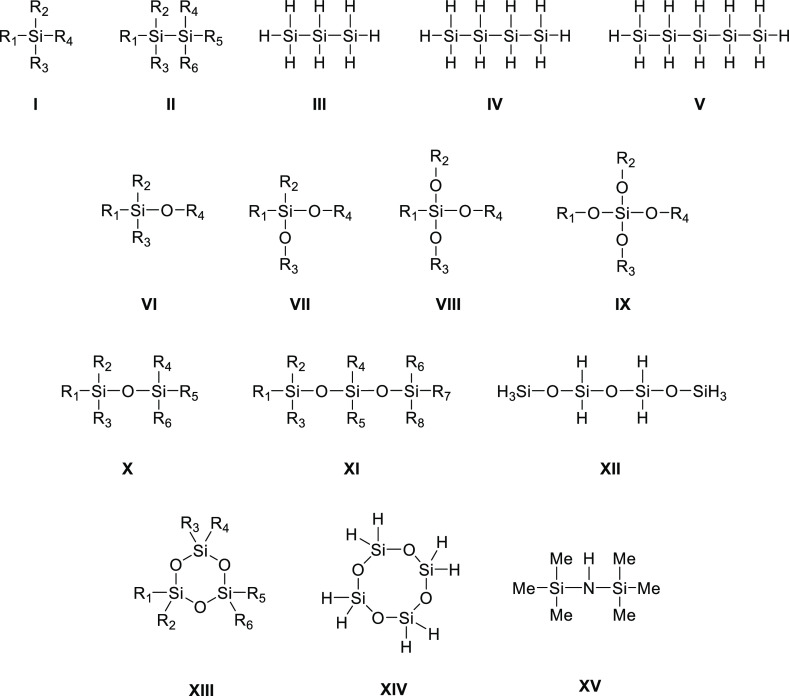
General Structures of Organosilicon Compounds **1**–**159** Considered in This Work and Their Division to Groups **I–XV** (R_1_–R_8_ = Alkyl, Alkenyl, Aryl, and/or Fluorine Substituents, See Table 1)

Following our previous work,^31^ the composite method W1X-1^26^ was used for the calculation of standard gas-phase enthalpies of formation (Δ_f_*H*_298K_^°^, kJ mol^–1^), entropies (*S*_298K_^°^, J K^–1^ mol^–1^), and heat capacities (*C*_*p*_, J K^–1^ mol^–1^) for 159 organosilicon compounds (Chart 1), which include 42 monosilanes (**1**–**42**, group **I**), 7 polysilanes (**43**–**49**, groups **II**–**V**), 31 silanols and alkoxysilanes (**50**–**80**, groups **VI**–**IX**), 70 acylic siloxanes (**81**–**150**, groups **X**–**XII**), 8 cyclic siloxanes (**151**–**158**, groups **XIII** and **XIV**), and 1 silylamine (**159**, group **XV**) with alkyl (Me = methyl, Et = ethyl, ^*i*^Pr = isopropyl, ^*s*^Bu = *sec*-butyl, and 3-Pe = 3-pentyl), alkenyl (Vi = vinyl), aryl (Ph = phenyl), and/or fluorine substituents. The size of the investigated systems was limited by the computational cost of the W1X-1 method that became prohibitive for molecules with more than ca. 35 atoms, requiring up to 10 TB of fast disk space for integral storage and weeks of wall-clock time even when the codes were executed in parallel. To this end, we chose to use the CBS-QB3 method^32^ for comparison purposes as it performed equally well with W1X-1 in our previous study on phosphines and phosphine oxides with only a fraction of the computational cost of W1X-1.

All structures were optimized with the Gaussian 16^33^ program package at the B3LYP^34−37^ level of theory using 6-311G(2d,d,p)^38,39^ (CBS-QB3) or cc-pV(T+d)Z^40,41^ (W1X-1) basis sets. For systems with multiple low-lying conformers, such as compounds with more than one ethyl or ethoxy substituent, extensive conformational scans were performed with the B3LYP/cc-pV(T+d)Z method to locate the global minimum on the potential energy surface. Total energies were computed for the lowest energy conformer of each molecule using the W1X-1^26^ and CBS-QB3^32^ protocols.

The CBS-QB3 method was used as implemented in the Gaussian 16 package.^33^ W1X-1 energies were obtained by the protocol of Chan and Radom using the Molpro code.^26,42−44^ Specifically, HF-CABS,^45^ CCSD-F12b,^46,47^ and CCSD(T)^48,49^ methods with cc-pVDZ-F12, cc-pVTZ-F12,^50−53^ aug′-cc-pV(D+d)Z, and aug′-cc-pV(T+d)Z^40,41^ basis sets were used to extrapolate three nonrelativistic energy components, *E*_HF-CABS_(cc-pVT/DZ-F12), *E*_ΔCCSD-F12b_(cc-pVT/DZ-F12), and *E*_Δ(T)_(aug′-cc-pV(T/D+d)Z), to the complete basis set (CBS) limit using the extrapolation formula *E*_L_ = *E*_CBS_ + *AL*^–α^,^54^ where *L* is the cardinal number of basis sets (2 or 3), and α is an adjustable parameter (5, 3.6725, and 2.0436 for HF-CABS, ΔCCSD, and Δ(T), respectively).^26^ The cc-pCVTZ basis set was used with FC-MP2 and DKH-MP2^55,56^ methods to obtain a combined core and scalar relativistic correlation term *E*_Δ(C+R)_ as a difference of the two single-point energies.^57,58^

For selected compounds, very high-level W2 energies were calculated with the Molpro code using the established procedure.^4,59^ The W2 method follows a similar protocol as W1X-1 with basis set extrapolation up to the pentuple-ζ level and without employing the F12 ansatz. Furthermore, CCSD(T)/MTsmall calculations, not MP2/cc-pCVTZ, are used to obtain the combined core and scalar relativistic correlation term.^4,48,49^ Because of the size of the systems in question, coupled cluster level geometry optimizations in W2 were replaced with density functional level calculations, as originally recommended by Martin and de Oliveira,^4^ using the B3LYP/cc-pV(T+d)Z combination augmented with Grimme’s empirical dispersion correction (GD3) with Becke–Johnson damping.^60,61^

For the determination of standard enthalpies of formation, heat capacities, and entropies, the density functional level harmonic vibrational frequencies were scaled with 0.985 (W1X-1 and W2) or 0.990 (CBS-QB3). The calculation of entropies and heat capacities was carried out within the rigid rotor-harmonic oscillator approximation and treating rotation modes involving single bonds as hindered rotors using the procedure implemented in Gaussian 16.^62^ A periodicity of 3 and a symmetry number of 3 were used for functional groups with local *C*_3_ symmetry, while 3 and 1 were used for other functional groups.

Standard gas-phase enthalpies of formation Δ_f_*H*_298K_^°^ were obtained using the atomization energy approach. For multiconformational molecules, the experimental enthalpy of formation reflects a Boltzmann distribution of conformers having statistically significant populations at 298 K. In contrast, our calculations use the most stable conformer for each molecule. This choice was made because Bolzmann averaging has been shown to lead to a correction that is similar in magnitude but opposite in sign to the correction for low-frequency internal rotations.^63^ Hence, both corrections should be treated on equal footing, that is, either included or omitted. Considering the number of compounds investigated in this study and the level of theory employed, the calculation of these correction terms would have been a prohibitively expensive task.

Reference values for the enthalpies of formation of gaseous atoms and thermal corrections for elements in their standard states were taken directly from the NIST/JANAF tables for elements H, C, N, O, and F.^64^ However, the commonly employed NIST/JANAF value for the standard enthalpy of formation of gaseous Si carries a very large uncertainty of 8.0 kJ mol^–1^, as opposed to elements H, C, O, and F, whose uncertainties are an order of magnitude smaller. For this reason, the theoretical W4 enthalpy of formation of gaseous Si, 452.71 kJ mol^–1^, reported by Karton and Martin was used as it has a statistical uncertainty of only 0.8 kJ mol^–1^.^65^ The atomization energies were also corrected for atomic spin–orbit (SO) coupling effects, a practice not uniformly followed in the field. While this correction can be obtained through theory, we chose to employ the experimental values tabulated by Moore.^66^

The computed W1X-1 thermochemical parameters were used to derive Benson group contributions for 60 silicon-based Benson groups and group pairs. The Benson group contributions were derived using a Convex Over and Under ENvelopes for Nonlinear Estimation (COUENNE) algorithm of the COIN-OR foundation^67^ implemented in OpenSolver^68,69^ and minimizing the squared differences between the computed thermochemical parameters and parameters calculated as sums of group contributions. Literature values were used for all carbon-based Benson groups, and entropy contributions were corrected for optical isomerism (*R* ln *n*, where *n* is the total number of stereoisomers) as well as internal (σ_int_) and external (σ_ext_) symmetries (−*R* ln σ_tot_, where σ_tot_ = σ_ext_Π^*i*^(σ_int_)_*i*_).^15^ When deriving group contribution values, the methyl repulsion correction term of Domalski and Hearing was used for tertiary carbon atoms,^10,11^ while ring strain was taken into account by using a single ring strain parameter for each ring size.^15^ Instead of using a single unsubstituted (parent) compound to determine the strain parameter for a given ring, it was optimized for all compounds of a particular ring type during the fitting procedure.

To obtain unique and well-converged sets of group contributions from the fits, the values of some groups must be fixed to avoid linear dependencies. In the case of element–carbon bonds, this has typically been achieved by setting the values of the group E–(C)(H)_3_ to be independent of element E and fixed to the value of C–(C)(H)_3_, as initially chosen by Benson.^9^ In the current case, this choice is not alone sufficient and the values of the group Si–(C)_3_(O) were set to match those of Si–(C)_4_, following the practice of Becerra and Walsh.^18^ Furthermore, the values of the group C_D_–(C_D_)(H)(Si) also needed to be fixed and were adjusted to be the same as those determined for C_D_–(C)(C_D_)(H). The fits obtained using this procedure reproduced the original W1X-1 thermochemical data excellently in the case of enthalpies (MAD 0.8 kJ mol^–1^, maximum deviation −6.5 kJ mol^–1^) and heat capacities (MAD 1.0 J K^–1^ mol^–1^, maximum deviation −10.6 J K^–1^ mol^–1^), while slightly poorer performance was seen in the case of entropies (MAD 3.9 J K^–1^ mol^–1^, maximum deviation 26.6 J K^–1^ mol^–1^).

## Results and Discussion

### Comparison of Calculated Gas-Phase Standard Enthalpies of Formation with Experimental Data

Before discussing the computational results (Table 1) in comparison with experimental data (Table 2), an analysis contrasting the W1X-1 values with those obtained with the CBS-QB3 method is warranted. Excluding data for the parent silane SiH_4_ and a few of its monoalkyl derivatives, the CBS-QB3 enthalpies for monosilanes **I** are always slightly greater than those obtained with the W1X-1 method, leading to a positive mean signed deviation (MSD) between the two data sets of 7 kJ mol^–1^. However, the opposite is true for all other groups. While the MSD values remain close to 0 for polysilanes **II**–**V** and silanols and alkoxysilanes **VI**–**IX**, −2 and −4 kJ mol^–1^, respectively, they are considerably more negative for acyclic (**X**–**XII**) and cyclic (**XII** and **XIV**) siloxanes, −14 and −13 kJ mol^–1^, respectively.

**Table 1:** Calculated Gas-Phase Standard Enthalpies of Formation (Δ_f_*H*_298K_^°^, kJ mol^–1^), Entropies (*S*_298K_^°^, J K^–1^ mol^–1^), and Heat Capacities (*C*_*p*_, J K^–1^ mol^–1^) of Monosilanes **1**–**42**, Polysilanes **43**–**49**, Silanols and Alkoxysilanes **50**–**80**, Acyclic Siloxanes **81**–**150**, Cyclic Siloxanes **151**–**158**, and Silylamine **159**^a^

			Δ_f_*H*° 298 K		*S*° 298 K	*C*_*p*_ 298 K	*C*_*p*_ 500 K	*C*_*p*_ 1000 K
group	molecule	chemical formula	W1X-1	CBS-QB3	W1X-1	W1X-1	W1X-1	W1X-1
**I**	**1**	SiH_4_	35.9	27.0	225.2	34.7	51.0	76.2
	**2**	SiH_3_Me	–23.8	–27.6	257.5	57.6	84.5	125.1
	**3**	SiH_3_Et	–32.8	–34.4	302.1	79.5	117.5	175.5
	**4**	SiH_3_Vi	96.9	94.3	288.5	68.5	100.6	146.6
	**5**	SiH_3_Ph	124.8	130.6	330.3	103.5	171.8	258.7
	**6**	SiH_3_^*i*^Pr	–52.7	–52.7	331.9	104.3	154.2	227.9
	**7**	SiH_3_^*s*^Bu	–72.3	–70.2	366.2	126.2	186.8	278.0
	**8**	SiH_3_(3-Pe)	–90.6	–86.8	402.4	144.8	218.5	328.1
	**9**	SiH_2_Me_2_	–85.9	–85.1	301.4	82.6	118.9	174.2
	**10**	SiH_2_EtMe	–94.8	–91.9	342.9	104.1	151.4	224.5
	**11**	SiH_2_MeVi	34.0	35.9	329.3	93.4	134.9	195.6
	**12**	SiH_2_MePh	63.3	70.5	389.9	133.0	210.5	312.0
	**13**	SiH_2_Me^*i*^Pr	–114.9	–110.7	372.0	129.0	188.1	276.7
	**14**	SiH_2_Me^*s*^Bu	–134.2	–128.0	406.5	150.7	220.4	326.7
	**15**	SiH_2_Me(3-Pe)	–151.7	–143.8	439.5	171.5	255.2	378.7
	**16**	SiH_2_Et_2_	–103.7	–98.8	373.0	125.8	183.9	274.7
	**17**	SiH_2_EtPh	52.7	62.9	426.8	154.2	242.6	362.1
	**18**	SiH_2_Vi_2_	153.5	156.6	344.5	105.1	151.6	217.3
	**19**	SiH_2_Ph_2_	210.3	223.4	462.3	184.9	302.5	449.8
	**20**	SiHMe_3_	–149.9	–145.2	337.6	109.3	153.9	223.5
	**21**	SiHEtMe_2_	–158.7	–152.1	382.2	130.2	186.0	273.6
	**22**	SiHMe_2_Vi	–30.6	–24.8	369.1	120.0	169.8	244.8
	**23**	SiHMe_2_Ph	–2.2	8.5	429.8	159.6	245.4	361.2
	**24**	SiHMe_2_^*i*^Pr	–178.7	–171.0	411.0	155.1	222.6	325.9
	**25**	SiHMe_2_^*s*^Bu	–197.9	–188.4	444.8	177.1	255.3	375.9
	**26**	SiHMe_2_(3-Pe)	–215.3	–204.3	477.9	197.8	289.8	427.7
	**27**	SiHEtMePh	–12.7	–0.8	461.4	182.6	279.0	411.9
	**28**	SiHMeVi_2_	88.2	95.1	388.9	131.6	186.3	266.3
	**29**	SiHMePhVi	115.7	127.2	448.5	170.9	261.6	382.6
	**30**	SiHVi_3_	207.8	215.4	411.8	141.2	201.4	287.4
	**31**	SiHPhVi_2_	234.9	247.5	468.4	184.0	278.7	404.3
	**32**	SiMe_4_	–215.0	–207.4	363.6	137.5	189.9	273.0
	**33**	SiEtMe_3_	–223.7	–214.4	420.2	157.8	221.5	323.0
	**34**	SiMe_3_Vi	–96.5	–87.6	406.5	147.7	205.2	294.1
	**35**	SiMe_3_Ph	–68.4	–55.2	463.4	189.0	281.9	410.8
	**36**	SiMe_2_Vi_2_	21.9	31.8	421.9	158.4	221.0	315.4
	**37**	SiEtMe_2_Ph	–78.8	–64.9	506.2	211.8	314.7	461.1
	**38**	SiMe_2_PhVi	49.1	63.2	485.6	199.5	297.6	432.0
	**39**	SiMe_2_Ph_2_	76.0	94.0	535.2	242.4	375.0	549.0
	**40**	SiMeVi_3_	139.9	150.1	438.8	170.3	237.8	337.0
	**41**	SiMePhVi_2_	166.1	180.4	503.7	212.1	314.5	453.7
	**42**	SiEt_4_	–251.9	–238.8	509.3	224.9	321.2	474.4
**II**	**43**	Si_2_H_6_	81.1	74.2	275.6	70.3	97.8	135.7
	**44**	Si_2_H_5_Me	22.9	20.1	331.6	94.5	131.5	184.6
	**45**	Si_2_H_4_Me_2_	–34.6	–33.4	367.0	118.5	165.2	233.5
	**46**	Si_2_Me_6_	–280.3	–267.3	513.5	227.2	305.9	430.5
**III**	**47**	Si_3_H_8_	120.4	113.7	350.3	105.8	144.0	194.9
**IV**	**48**	Si_4_H_10_	158.4	151.7	415.4	141.9	190.4	254.2
**V**	**49**	Si_5_H_12_	196.1	189.4	481.1	177.7	236.7	313.4
**VI**	**50**	SiH_3_OH	–280.1	–286.7	256.8	46.1	66.8	93.0
	**51**	SiH_2_MeOH	–350.7	–352.4	297.6	70.7	101.0	142.1
	**52**	SiH_2_EtOH	–359.3	–359.3	332.2	93.3	134.4	192.7
	**53**	SiHMe_2_OH	–419.7	–417.7	337.4	97.5	136.2	191.4
	**54**	SiMe_3_OH	–488.2	–483.4	375.9	125.0	171.6	240.7
	**55**	SiH_3_OMe	–245.8	–253.7	300.2	63.1	93.4	141.3
	**56**	SiH_2_Me(OMe)	–316.4	–319.7	340.7	87.8	127.5	190.4
	**57**	SiHMe_2_(OMe)	–385.0	–384.9	380.3	115.0	162.9	239.7
**VII**	**58**	SiH_2_(OH)_2_	–628.7	–633.2	280.0	62.9	85.1	110.3
	**59**	SiH_2_(OMe)_2_	–557.7	–565.7	365.7	92.1	136.0	206.2
	**60**	SiHMe(OMe)_2_	–630.3	–635.1	404.2	115.0	169.4	255.0
	**61**	SiHVi(OMe)_2_	–509.5	–513.6	430.8	128.7	186.5	276.6
	**62**	SiHPh(OMe)_2_	–483.3	–482.7	487.2	171.2	263.3	393.3
	**63**	SiMe_2_(OMe)_2_	–702.3	–705.1	443.3	147.1	207.0	305.0
	**64**	SiMeVi(OMe)_2_	–582.4	–584.6	469.1	156.0	221.7	325.9
	**65**	SiMePh(OMe)_2_	–556.8	–555.0	524.0	200.6	301.9	446.5
	**66**	SiVi_2_(OMe)_2_	–462.6	–464.3	480.4	174.8	246.6	355.8
	**67**	SiPhVi(OMe)_2_	–437.4	–435.4	543.3	225.6	335.8	487.8
	**68**	SiPh_2_(OMe)_2_	–412.4	–406.9	597.6	250.5	390.7	580.4
**VIII**	**69**	SiH(OH)_3_	–985.9	–988.7	316.2	79.1	103.3	127.7
	**70**	SiMe(OMe)_2_OH	–986.6	–992.3	440.7	137.1	190.0	273.0
	**71**	SiEt(OMe)_2_OH	–994.3	–999.1	476.2	156.6	220.2	322.2
	**72**	SiMe(OMe)_3_	–948.6	–957.0	480.5	162.0	227.1	333.1
	**73**	SiEt(OMe)_3_	–956.7	–964.3	515.4	173.0	247.6	371.0
**IX**	**74**	Si(OH)_4_	–1341.7	–1344.2	335.9	98.2	122.7	145.5
	**75**	Si(OMe)_3_OH	–1232.3	–1243.4	466.8	143.3	199.3	289.1
	**76**	Si(OEt)(OMe)_2_OH	–1267.5	–1277.0	501.6	174.1	243.2	349.4
	**77**	Si(OEt)_2_(OMe)OH	–1302.8	–1310.8	533.2	199.1	280.2	401.7
	**78**	Si(OMe)_4_	–1195.8	–1209.9	503.1	162.9	227.2	337.7
	**79**	Si(OEt) (OMe)_3_	–1231.3	–1243.8	541.8	184.5	262.1	389.4
	**80**	Si(OEt)_4_	–1337.7	–1345.7	636.2	252.8	369.2	545.4
**X**	**81**	O(SiH_3_)_2_	–339.7	–356.3	316.2	71.3	106.4	152.3
	**82**	O(SiMe_3_)(SiH_3_)	–550.7	–556.8	436.3	151.4	211.9	300.3
	**83**	O(SiF_3_)(SiH_3_)	–1605.9	–1620.9	383.3	99.0	131.6	166.2
	**84**	O(SiH_2_Me)(SiH_3_)	–410.7	–422.3	355.9	96.1	140.8	201.5
	**85**	O(SiH_2_Vi)(SiH_3_)	–289.3	–300.1	376.2	106.7	156.7	222.8
	**86**	O(SiH_2_Ph)(SiH_3_)	–259.6	–265.8	435.5	146.5	232.3	339.2
	**87**	O(SiH_2_F)(SiH_3_)	–759.8	–774.9	338.8	78.7	114.1	156.6
	**88**	O(SiHMe_2_)(SiH_3_)	–481.0	–489.3	403.8	123.6	176.4	251.0
	**89**	O(SiHVi_2_)(SiH_3_)	–241.3	–248.0	438.2	145.3	208.6	293.7
	**90**	O(SiHF_2_)(SiH_3_)	–1190.3	–1204.1	358.2	88.3	122.7	161.2
	**91**	O(SiHMePh)(SiH_3_)	–332.7	–335.7	485.4	174.0	267.9	388.6
	**92**	O(SiH_2_Me)_2_	–481.4	–488.3	394.0	121.7	175.6	250.8
	**93**	O(SiHMe_2_)(SiH_2_Me)	–550.7	–555.1	439.7	149.1	211.1	300.3
	**94**	O(SiH_2_Ph)(SiH_2_Me)	–329.9	–330.6	476.2	171.7	266.9	388.4
	**95**	O(SiMe_3_)(SiH_2_Me)	–621.2	–622.5	478.1	176.9	246.7	349.6
	**96**	O(SiHMe_2_)_2_	–621.6	–621.7	486.5	176.5	246.7	349.7
	**97**	O(SiMe_3_)(SiHMe_2_)	–690.8	–689.1	533.2	204.3	282.2	399.0
	**98**	O(SiMe_3_)_2_	–760.0	–756.2	559.0	232.9	318.3	448.5
	**99**	O(SiH_2_Vi)_2_	–238.9	–244.5	440.4	142.6	207.2	293.4
	**100**	O(SiH_2_F)_2_	–1179.1	–1192.1	343.0	86.1	121.7	160.9
	**101**	O(SiHF_2_)(SiH_2_F)	–1607.7	–1619.7	380.9	96.0	130.4	165.6
	**102**	O(SiF_3_)(SiH_2_F)	–2022.4	–2035.4	394.9	106.4	139.3	170.6
	**103**	O(SiHF_2_)_2_	–2034.6	–2045.5	393.4	105.5	139.0	170.4
	**104**	O(SiF_3_)(SiHF_2_)	–2448.8	–2460.7	413.2	116.0	147.8	175.4
	**105**	O(SiF_3_)_2_	–2861.4	–2874.1	461.3	130.7	160.9	184.5
**XI**	**106**	SiH_2_(OSiH_3_)_2_	–746.1	–771.7	412.6	110.5	163.2	228.7
	**107**	SiH_2_(OSiH_2_Me)(OSiH_3_)	–816.3	–838.0	459.5	135.9	197.9	278.0
	**108**	SiH_2_(OSiH_2_Vi)(OSiH_3_)	–695.9	–716.0	476.5	147.0	214.1	299.4
	**109**	SiH_2_(OSiH_2_Ph)(OSiH_3_)	–665.7	–680.3	559.8	202.8	305.9	432.2
	**110**	SiH_2_(OSiH_2_F)(OSiH_3_)	–1166.0	–1190.8	428.9	118.0	170.9	233.0
	**111**	SiH_2_(OSiMe_3_)(OSiH_3_)	–958.5	–973.7	532.7	191.0	268.9	376.8
	**112**	SiH_2_(OSiHMe_2_)(OSiH_3_)	–888.5	–905.8	489.9	163.0	233.2	327.4
	**113**	SiH_2_(OSiHF_2_)(OSiH_3_)	–1596.1	–1620.1	446.1	127.7	179.6	237.7
	**114**	SiH_2_(OSiF_3_)(OSiH_3_)	–2011.9	–2036.7	464.9	138.4	188.5	242.7
	**115**	SiH_2_(OSiH_2_Me)_2_	–888.2	–904.2	500.4	161.6	232.7	327.3
	**116**	SiH_2_(OSiHMe_2_)(OSiH_2_Me)	–959.5	–972.0	549.7	188.8	268.1	376.7
	**117**	SiH_2_(OSiMe_3_)(OSiH_2_Me)	–1029.4	–1039.9	568.5	216.6	303.7	426.1
	**118**	SiH_2_(OSiH_2_F)_2_	–1585.0	–1607.1	445.5	125.8	178.6	237.3
	**119**	SiH_2_(OSiHMe_2_)_2_	–1030.3	–1039.4	567.5	216.0	303.5	426.1
	**120**	SiH_2_(OSiMe_3_)(OSiHMe_2_)	–1100.2	–1107.2	627.0	244.0	339.2	475.5
	**121**	SiH_2_(OSiMe_3_)_2_	–1169.9	–1175.1	648.2	272.3	375.1	524.9
	**122**	SiHMe(OSiH_3_)_2_	–821.1	–843.2	460.5	137.5	198.5	278.1
	**123**	SiHVi(OSiH_3_)_2_	–699.2	–720.8	468.0	147.1	213.7	299.2
	**124**	SiHPh(OSiH_3_)_2_	–671.9	–689.0	530.4	188.5	290.3	415.9
	**125**	SiHF(OSiH_3_)_2_	–1178.3	–1203.6	433.8	120.3	171.9	233.3
	**126**	SiHMe(OSiH_2_Me)(OSiH_3_)	–892.1	–909.5	498.1	162.8	233.1	327.4
	**127**	SiHMe(OSiHMe_2_)(OSiH_3_)	–963.4	–977.0	526.2	190.3	268.7	376.9
	**128**	SiHMe(OSiMe_3_)(OSiH_3_)	–1033.0	–1045.1	572.5	218.3	304.3	426.2
	**129**	SiHMe(OSiH_2_Me)_2_	–962.9	–975.5	531.7	188.5	268.0	376.7
	**130**	SiHMe(OSiHMe_2_)(OSiH_2_Me)	–1034.4	–1043.4	566.6	215.9	303.5	426.2
	**131**	SiHMe(OSiMe_3_)(OSiH_2_Me)	–1104.1	–1111.3	604.3	243.9	339.1	475.5
	**132**	SiHMe(OSiHMe_2_)_2_	–1104.4	–1110.6	619.7	243.9	339.2	475.6
	**133**	SiHMe(OSiMe_3_)(OSiHMe_2_)	–1174.7	–1179.1	644.2	272.7	377.5	528.8
	**134**	SiHMe(OSiMe_3_)_2_	–1243.8	–1246.5	702.0	304.1	417.0	582.2
	**135**	SiHF(OSiH_2_F)(OSiH_3_)	–1594.8	–1622.9	454.0	136.3	188.1	246.0
	**136**	SiHF(OSiHF_2_)(OSiH_3_)	–2027.1	–2051.1	464.9	137.6	188.3	242.3
	**137**	SiMe_2_(OSiH_3_)_2_	–894.7	–915.5	492.1	165.7	234.3	327.6
	**138**	SiMe_2_(OSiH_2_Me)(OSiH_3_)	–965.5	–981.5	529.3	191.1	269.0	376.9
	**139**	SiMe_2_(OSiHMe_2_)(OSiH_3_)	–1036.6	–1049.1	571.8	222.8	308.8	430.5
	**140**	SiMe_2_(OSiMe_3_)(OSiH_3_)	–1106.2	–1116.9	620.1	251.6	345.0	480.1
	**141**	SiMe_2_(OSiH_2_Me)_2_	–1036.0	–1047.4	562.1	216.8	303.8	426.2
	**142**	SiMe_2_(OSiHMe_2_)(OSiH_2_Me)	–1107.2	–1115.0	625.4	245.0	339.7	475.8
	**143**	SiMe_2_(OSiMe_3_)(OSiH_2_Me)	–1176.7	–1182.8	641.8	273.3	375.7	525.2
	**144**	SiMe_2_(OSiMe_3_)_2_	–1316.6	–1317.9	718.3	329.0	447.2	624.1
	**145**	SiMe_2_(OSiHMe_2_)_2_	–1177.1	–1182.3	656.1	272.0	375.2	525.2
	**146**	SiMe_2_(OSiMe_3_)(OSiHMe_2_)	–1246.9	–1250.4	683.3	300.9	411.3	574.7
	**147**	SiF_2_(OSiH_3_)_2_	–1598.9	–1625.4	436.9	131.1	181.0	238.2
	**148**	SiF_2_(OSiH_2_F)(OSiH_3_)	–2017.1	–2043.8	461.6	138.7	188.7	242.6
	**149**	SiF_2_(OSiH_2_F)_2_	–2434.9	–2460.5	477.5	146.2	196.4	247.0
**XII**	**150**	O(SiH_2_OSiH_3_)_2_	–1151.9	–1186.1	494.3	150.0	220.1	305.1
**XIII**	**151**	(OSiH_2_)_3_	–1196.3	–1215.7	349.9	116.9	170.9	229.6
	**152**	(OSiHMe)(OSiH_2_)_2_	–1273.4	–1290.0	404.3	143.9	206.1	279.0
	**153**	(OSiMe_2_)(OSiH_2_)_2_	–1348.1	–1362.9	437.4	171.9	241.8	328.4
	**154**	(OSiHMe)_2_(OSiH_2_)	–1350.2	–1363.8	438.1	171.0	241.4	328.4
	**155**	(OSiMe_2_)(OSiHMe)(OSiH_2_)	–1424.6	–1436.6	482.2	199.0	277.1	377.9
	**156**	(OSiHMe)_3_	–1426.6	–1437.3	482.8	198.2	276.8	377.9
	**157**	(OSiMe_2_)_3_	–1648.3	–1653.8	583.4	282.5	384.0	526.2
**XIV**	**158**	(OSiH_2_)_4_	–1623.5	–1656.2	467.6	165.7	235.7	314.0
**XV**	**159**	NH(SiMe_3_)_2_	–472.0	–454.0	551.0	247.8	334.3	466.3

a Used abbreviations: Me = methyl, Et = ethyl, ^*i*^Pr = isopropyl, ^*s*^Bu = *sec*-butyl, 3-Pe = 3-pentyl, Vi = vinyl, and Ph = phenyl.

**Table 2:** Experimental (Exptl.) and Calculated (CBS-QB3, W1X-1, and W2) Gas-Phase Standard Enthalpies of Formation (Δ_f_*H*_298K_^°^, kJ mol^–1^) of Silicon Compounds Considered in This Work^a^

	Δ_f_*H*° 298 K			
molecule	exptl.	CBS-QB3	W1X-1	W2
SiH_4_	34.3 ± 1.2	27.0	35.9	
Si_2_H_6_	79.9 ± 1.5	74.2	81.1	
Si_3_H_6_	120.9 ± 4.4	113.7	120.4	
SiH_3_Me	–29.1 ± 4.0	–27.6	–23.8	
SiH_2_Me_2_	–94.7 ± 4.0	–85.1	–85.9	
SiHMe_3_	–163.4 ± 4.0	–145.2	–149.9	
SiMe_4_	–233.2 ± 3.2	–207.4	–215.0	–212.8
Si_2_Me_6_	–303.7 ± 5.5	–267.3	–280.3	–277.0
Si(OH)_4_	–1351.3 ± 1.7	–1344.2	–1341.7	
SiMe_3_(OH)	–500.0 ± 3.0	–483.4	–488.2	
Si(OEt)_4_	–1356.0 ± 6.0	–1345.7	–1337.7	–1331.4
O(SiMe_3_)_2_	–777.4 ± 6.0	–756.2	–760.0	–761.0
NH(SiMe_3_)_2_	–477.0 ± 5.0	–454.0	–472.0	–460.8

a Experimental data are taken from the two most recent compilations by Becerra and Walsh.^18,19^

Closer inspection of data in Table 1 reveals that the observed trends originate from systematic differences between W1X-1 and CBS-QB3 results. For example, the CBS-QB3 enthalpy of formation of the parent silane SiH_4_ is less than the corresponding W1X-1 prediction, and each successive substitution by alkyl, alkenyl, or aryl groups affects the difference in a very consistent way. Thus, the CBS-QB3 enthalpies become greater than W1X-1 values for monosilanes with two or more substituents, and the differences are notable for tetrasubstituted species and for systems with more than one phenyl substituent. Similarly, the CBS-QB3 enthalpies for siloxanes with one or two silyl or fluorosilyl substituents are markedly lower than the corresponding W1X-1 values, and the prevalence of this type of compounds in groups **X** and **XI** manifests itself in the very negative MSD value.

Having established that there are systematic differences between the two sets of computational standard gas-phase enthalpies of formation, an important question to ask is which method, W1X-1 or CBS-QB3, is more trustworthy, and how do the calculated numbers compare with their experimental counterparts. From a purely theoretical viewpoint, W1X-1 is more robust and advanced than CBS-QB3 and should be preferred. This is also borne out by comparing the 1/2/3σ confidence intervals of CBS-QB3 (determined against the active thermochemical tables), ±7/±14/±21 kJ mol^–1^,^70^ to those of W1X-1 (estimated from the MAD with respect to G2 and G3 data sets), ±3/±6/±9 kJ mol^–1^.^26^ Since both CBS-QB3 and W1X-1 contain empirical parameters that are potential sources of systematic error, we used the parameter-free W2 method as a very high-level reference in cases where significant (>3σ) discrepancies between calculated and experimental enthalpies were observed. Even though the confidence intervals of W2 have not been determined, its MAD with respect to G2 enthalpies of formation is lower than the average 2σ uncertainty of experimental values in the reference data set.^4,59^

As discussed in the Introduction section, reliably determined gas-phase standard enthalpies of formation for silicon compounds are few and far between, which is reflected in the reference data available for comparison with the values calculated herein.^17−19^ Well-established experimental values exist only for 13 compounds in Chart 1 (Table 2; reported uncertainties correspond to 2σ confidence intervals). The two most recent compilations by Becerra and Walsh contain the citations to the original work as well as an in-depth discussion of the reliability of the data and why particular values are recommended over others.^18,19^ Becerra and Walsh have also determined enthalpies of formation via semi-empirical means (bond and group additivity considerations as well as electronegativity correlations) that can also be used for comparison.^17−19^ These are of lesser significance than first-hand (calorimetric) measurements, for which reason we have explicitly pointed out their use in the following discussion. The same is also true for the data reported by Voronkov et al.^20−25^ that have consistently been flagged dubious by Becerra and Walsh through comparisons with other reference data or with estimates based on reasonable chemical expectations.^17−19^

Comparison of computational data for parent mono- and polysilanes with the recommended experimental values shows that the W1X-1-calculated enthalpies of formation for silane SiH_4_, disilane Si_2_H_6_, and trisilane Si_3_H_8_ (35.9, 81.1, and 120.4 kJ mol^–1^, respectively) are in excellent agreement with calorimetric data, 34.3 ± 1.2, 79.9 ± 1.5, and 120.9 ± 4.4 kJ mol^–1^, respectively. In comparison, the CBS-QB3 calculated enthalpies for the same set (27.0, 74.2, and 113.7 kJ mol^–1^, respectively) are all less endothermic and further away from the experimental values.

The well-established experimental enthalpies of formation for the methylsilane series SiH_3_Me, SiH_2_Me_2_, SiHMe_3_, and SiMe_4_ are −29.1 ± 4.0, −94.7 ± 4.0, −163.4 ± 4.0, and −233.2 ± 3.2 kJ mol^–1^, respectively. Additionally, Voronkov et al. have reported a value of −229.0 ± 3.0 kJ mol^–1^ for SiMe_4_,^20^ in good harmony with the earlier calorimetric measurement. A comparison of these data with W1X-1 (−23.8, −85.9, −149.9, and −215.0 kJ mol^–1^, respectively) and CBS-QB3 (−27.6, −85.1, −145.2, and −207.4 kJ mol, respectively) values shows that the difference between calculated and experimental values increases with the number of methyl groups. In fact, both W1X-1 and CBS-QB3 values for SiMe_4_ are statistically (3σ) different than the experimental result. The W2 method gives −212.8 kJ mol^–1^ for the enthalpy of formation of SiMe_4_, in excellent agreement with the W1X-1 value. For this reason, we conclude that the experimental enthalpy of formation of SiMe_4_ is too exothermic. Furthermore, since the experimental enthalpies of formation for SiH_3_Me, SiH_2_Me_2_, and SiHMe_3_ are based on data from methyl redistribution reactions and employ the calorimetric enthalpy of formation of SiMe_4_ as a common reference,^71^ their values should also be adjusted accordingly.

The recommended enthalpy of formation of hexamethyldisilane Si_2_Me_6_, −303.7 ± 5.5 kJ mol^–1^, has been determined using solution calorimetry. This value is statistically (3σ) different from those obtained with W1X-1 and CBS-QB3 methods (−280.3 and −267.3 kJ mol^–1^, respectively). For comparison, the W2 method yields −277.0 kJ mol^–1^, in excellent agreement with W1X-1. Consequently, the experimental enthalpy of formation of Si_2_Me_6_ is almost certainly too exothermic.

Voronkov et al. have reported a value of −297.0 ± 5.0 kJ mol^–1^ for the enthalpy of formation of tetraethylsilane SiEt_4_.^20^ This result is significantly more exothermic than the values calculated with W1X-1 and CBS-QB3 (−251.9 and −238.8 kJ mol^–1^, respectively) and clearly in error. In contrast, Becerra and Walsh have recently suggested a value of −269 kJ mol^–1^ for this quantity based on group additivity estimates,^19^ in much better agreement with the calculated enthalpies and the W1X-1 value in particular. In a similar fashion, the estimated enthalpies of formation for the ethylsilane series SiH_3_Et, SiH_2_Et_2_, and SiHEt_3_ are −46, −129, and −214 kJ mol^–1^, respectively.^18^ However, these are based on an older methyl-to-ethyl substitution replacement enthalpy, ΔΔ(Me/Et) = −17 kJ mol^–1^, whereas a revised value of −9 kJ mol^–1^ was used to derive the estimate for SiEt_4_.^19^ Correcting the ethylsilane data with the revised ΔΔ(Me/Et) value, which, in fact, matches perfectly with the difference between the W1X-1 enthalpies for SiH_3_Me and SiH_3_Et (Table 1), gives −38, −113, and −190 kJ mol^–1^ for SiH_3_Et, SiH_2_Et_2_, and SiHEt_3_, respectively. Considering the large 2σ uncertainty (±16 kJ mol^–1^) associated with these estimations, the agreement with our W1X-1 values for SiH_3_Et and SiH_2_Et_2_ (−32.8 and −103.7 kJ mol^–1^, respectively) is very good.

Voronkov et al. have quoted −191.0 ± 5.0 kJ mol^–1^ for the enthalpy of formation of trimethylvinylsilane SiMe_3_Vi.^20^ This value has been heavily criticized by Walsh and Becerra,^17,18^ and both W1X-1 and CBS-QB3 results obtained herein (−96.5 and −87.6 kJ mol^–1^, respectively) clearly support these concerns. A revised value of −125 kJ mol^–1^ has been proposed by Becerra and Walsh based on hydrogenation enthalpies and isodesmic reaction data.^18^ Even though this result is in better agreement with the calculated data than the value reported by Voronkov et al., the estimated enthalpy is, nevertheless, too exothermic based on our calculated values. Becerra and Walsh have also derived a recommended value for the enthalpy of formation of the parent vinylsilane SiH_3_Vi, 87.0 kJ mol^–1^,^19^ that is in reasonably good agreement with our W1X-1 and CBS-QB3 enthalpies (96.9 and 94.3 kJ mol^–1^, respectively). We note that if the estimate of Becerra and Walsh for SiH_3_Vi is corrected with ΔΔ(H/Me) = −63 kJ mol^–1^, determined from our data for the vinylsilane series, the estimated enthalpy of formation for SiMe_3_Vi becomes −102 kJ mol^–1^, in good agreement with our calculations.

The benchmark enthalpies of formation reported for tetrahydroxysilane Si(OH)_4_, trimethylsilanol SiMe_3_(OH), and tetraethoxysilane Si(OEt)_4_ are −1351.3 ± 1.7, −500.0 ± 3.0, and −1356.0 ± 6.0 kJ mol^–1^, respectively. Our W1X-1 (−1341.7, −488.2, and −1337.7 kJ mol^–1^, respectively) and CBS-QB3 (−1344.2, −483.4, and −1345.7 kJ mol^–1^, respectively) results are mostly in harmony with each other and in reasonable agreement with the experimental values. The only exception to the trend is tetraethoxysilane, for which the W1X-1 enthalpy hits the limits of the associated 3σ confidence intervals. The W2 enthalpy of Si(OEt)_4_ is −1331.4 kJ mol^–1^, and, therefore, in better agreement with the W1X-1 value than with experimental data, suggesting that the latter should be slightly adjusted. Voronkov et al. have reported an even less exothermic enthalpy of formation for this compound, −1315.0 ± 6.0 kJ mol^–1^, that is clearly erroneous, but the value they quote for trimethoxymethylsilane SiMe(OMe)_3_, −944.0 ± 5.0 kJ mol^–1^, is in very good agreement with our W1X-1 and CBS-QB3 results (−948.6 and −957.6 kJ mol^–1^, respectively).^20^

Using group additivity approaches, Becerra and Walsh have derived enthalpies of formation of −259 and −1220 kJ mol^–1^ for methoxysilane SiH_3_(OMe) and tetramethoxysilane Si(OMe)_4_, respectively.^19^ These are in good agreement with our CBS-QB3 data (−253.7 and −1209.9 kJ mol^–1^, respectively) but differ more from the values calculated with W1X-1 (−245.8 and −1195.9 kJ mol^–1^, respectively). The match between group additivity estimates and CBS-QB3 data is expected to be only fortuitous, and the calculated W1X-1 values should be considered the most trustworthy of the three. An additional reference point is provided by Voronkov et al., who quote −1180.0 ± 5.0 kJ mol^–1^ for the enthalpy of formation of tetramethoxysilane,^20^ in reasonable agreement with our W1X-1 result.

Only a single well-established (bomb calorimetry) enthalpy of formation has been reported for siloxanes considered in this work: −777.4 ± 6.0 kJ mol^–1^ for hexamethyldisiloxane O(SiMe_3_)_2_. A reassessment of this value by Voronkov et al. led to a matching result of −778.6 ± 4.0 kJ mol^–1^.^22^ The calculated W1X-1 and CBS-QB3 enthalpies of formation are both less exothermic (−760.0 and −756.2 kJ mol^–1^, respectively), and the W1X-1 value is only barely inside the associated 3σ confidence intervals. A reassessment of the enthalpy of formation of hexamethyldisiloxane with the W2 method yields −761.0 kJ mol^–1^, in excellent agreement with the W1X-1 value. This suggests that the experimental data are most likely slightly too exothermic.

Voronkov et al. have also determined the standard enthalpy of formation of hexamethylcyclotrisiloxane (OSiMe_2_)_3_, −1568.0 ± 10.0 kJ mol^–1^.^24^ Their result is almost 100 kJ mol^–1^ less exothermic than our W1X-1 and CBS-QB3 enthalpies that are in excellent agreement with each other (−1648.3 and −1653.8 kJ mol^–1^, respectively), casting further doubt over the experimental work of Voronkov et al.

The last compound to consider is hexamethyldisilazane NH(SiMe_3_)_2_, for which the recommended enthalpy of formation, −477.0 ± 5.0 kJ mol^–1^, is based on solution calorimetry. A more recent investigation was performed by Voronkov et al., leading to a slightly less exothermic value, −450.8 ± 10.0 kJ mol^–1^,^25^ but with much larger uncertainty. Interestingly, our calculated W1X-1 enthalpy (−472.0 kJ mol^–1^) is a good match with the result from solution calorimetry, whereas the CBS-QB3 value (−454.0 kJ mol^–1^) agrees nicely with the work of Voronkov et al. Consequently, we used the W2 method as an adjudicator, and the result, −460.8 kJ mol^–1^, agrees slightly better with the CBS-QB3 data, casting some doubt over the use of solution calorimetry result as the well-established experimental value.

Considered as a whole, the standard gas-phase enthalpies of formation calculated with the W1X-1 and W2 methods are consistently in better agreement with experimental data than those obtained with the CBS-QB3 approach. Consequently, systematic differences between W1X-1 and CBS-QB3 can be attributed to inadequate treatment of electron correlation effects in the latter that become more prominent with increasing molecular size. This is in stark contrast to our previous study on phosphines and phosphine oxides,^31^ in which case W1X-1 and CBS-QB3 showed much more uniform performance, albeit for a more limited set of compounds with less variety in the employed substituents. W1X-1 enthalpies are, therefore, considered superior to CBS-QB3 results and used exclusively in the remaining parts of the analysis and discussion. Furthermore, in those cases where W1X-1 and experimental values differ more than the associated 3σ intervals, the very high-level W2 method yields values in better agreement with W1X-1. This allows us to conclude that the experimental standard gas-phase enthalpies of formation of SiMe_4_ and Si_2_Me_6_ are too exothermic, while those of Si(OEt)_4_, O(SiMe_3_)_2_, and NH(SiMe_3_)_2_ are borderline cases and could also require adjustment.

### Comparison of Calculated Gas-Phase Standard Enthalpies of Formation with Prior Computational Data

To the best of our knowledge, the works of Burcat and Goos^28^ and Janbazi et al.^29,30^ represent the most recent large-scale attempts to calculate thermochemical parameters of organosilicon compounds using computational methods. Their data have been obtained with the G3 and G4 composite methods, respectively, whose expected accuracy is in between those of W1X-1 and CBS-QB3, although closer to the former than the latter.^70^ The earlier work of Feller and Dixon,^27^ while not nearly as comprehensive, needs to be mentioned in this context because it reports very high-level CCSD(T)/CBS benchmark data for nine small silicon compounds, including SiH_4_ and Si_2_H_6_. We stress that the abovementioned papers are not by any means the only ones dealing with computational thermochemistry of organosilicon compounds and many other authors have touched different aspects of the field over the years. Regardless, the efforts by Burcat and Goos^28^ and Janbazi et al.^29,30^ are the most comprehensive available and cover a large part of the species that had been investigated prior to their work. For a review of pre-2015 computational data on the field, the papers by Burcat and Goos^28^ and Becerra and Walsh^19^ are excellent references.

A comparison of computational data for Si_x_H_y_ systems shows that our W1X-1 values for SiH_4_, Si_2_H_6_, and Si_3_H_8_ (35.9, 81.1, and 120.4 kJ mol^–1^, respectively) are identical, within the accuracy of the methods, to prior results of Feller and Dixon and Burcat and Goos after adjusting the latter values to the same temperature (298.15 K) and employing the same atomic reference values including spin–orbit corrections (adjusted values 33.0, 76.3, and 122.7 kJ mol^–1^ for SiH_4_,^27^ Si_2_H_6_,^27^ and Si_3_H_8_,^28^ respectively). The G4 enthalpies of formation given by Janbazi et al.^29^ for Si_2_H_6_ and Si_3_H_8_ agree with the above values after similar adjustments (78.8 and 118.4 kJ mol^–1^, respectively). Interestingly, the same does not hold for the methylsilane series, for which the adjusted data from Janbazi et al.^29^ (−26.2, −87.3, −160.0, and −233.6 kJ mol^–1^ for SiH_3_Me, SiH_2_Me_2_, SiHMe_3_, and SiMe_4_, respectively) show a gradually increasing deviation from our W1X-1 values (−23.8, −85.9, −149.9, and −215.0 kJ mol^–1^, respectively). For comparison, the adjusted G3 values of Burcat and Goos^28^ for SiHMe_3_ and SiMe_4_ are −157.7 and −223.8 kJ mol^–1^, respectively. Most surprising are, however, the CBS-QB3 values of Janbazi et al.^29^ that are, after adjustments, 69.2, 107.3, and −213.8 kJ mol^–1^ for Si_2_H_6_, Si_3_H_8_, and SiMe_4_, respectively, and differ from the values reported by us (74.2, 113.7, and −207.4 kJ mol^–1^), even though the computational method used is identical. It is unfortunate that no more details of their work are provided by Janbazi et al. as this would have allowed us to trace the origin of this discrepancy.

Even more perplexing is the observation that the G4 data reported by Janbazi et al.^30^ for silanols and alkoxysilanes in their second paper are in much better agreement with our values in Table 1 than what was seen in the case of simple (alkyl)silanes. This is surprising, given that the same composite method and atomization approach were used in both studies. Thus, we have no significant reservations about the enthalpies of formation reported in the follow-up work of Janbazi et al.^30^ saved for the fact that their reference value for SiH_3_OH, −285.2 kJ mol^–1^, can be slightly too exothermic (cf. W1X-1 value of −280.1 kJ mol^–1^). If this turns out to be the case, a significant systematic error could occur when the value is combined with large stoichiometric coefficients used to calculate the standard enthalpies of formation via isodesmic reactions. We will return to the computational results of Janbazi et al. when discussing the group additivity contributions they have determined based on the reported enthalpies.

### Benson Thermochemical Group Contributions for Silicon and Their Use in Assessing the Reliability of Experimental Data Reported by Voronkov et al.

Group additivity contributions allow for fast and accurate estimation of chemical properties of many organic compounds. In this work, we used the calculated W1X-1 thermochemical data in Table 1 to derive Benson group contributions for 60 silicon-based groups and group pairs given in Tables 3 and 4, respectively. The convention by Holmes and Aubry was adopted, where all values are rounded to the nearest integer to underline the internal character of group contribution methods to estimate, rather than calculate, thermochemical parameters.^13,14^ In the case of aryl-substituted species, Benson groups always occur in pairs, which prevents the easy assignment of unambiguous values for individual groups.^72^ These can be derived by assigning arbitrary reference values for key groups, such as the group C_B_–(C_B_)_2_(Si) discussed herein. While this convention has been adopted by some authors, including Benson in his later works,^15^ we chose to report group pair contributions following the practice adopted in our previous work.^31^

**Table 3:** Thermochemical Benson Group Contributions for Standard Enthalpies of Formation (Δ_f_*H*_298K_^°^, kJ mol^–1^), Entropies (*S*_298K_^°^, J K^–1^ mol^–1^), and Heat Capacities (*C*_*p*_, J K^–1^ mol^–1^) Derived from the Results of W1X-1 Calculations

Benson group	Δ_f_*H*° 298 K	*S*° 298 K	*C*_*p*_ 298 K	*C*_*p*_ 500 K	*C*_*p*_ 1000 K
Si–(C)(H)_3_	19	156	32	45	63
Si–(C_D_)(H)_3_	34	149	28	45	64
Si–(H)_3_(O)	38	151	30	44	63
Si–(H)_3_(Si)	41	152	35	49	68
Si–(C)_2_(H)_2_	–1	72	31	40	51
Si–(C_D_)_2_(H)_2_	28	53	25	40	52
Si–(O)_2_(H)_2_	9	56	31	41	51
Si–(Si)_2_(H)_2_	38	68	36	46	59
Si–(C)(C_D_)(H)_2_	14	63	28	40	51
Si–(C)(H)_2_(O)	10	63	30	39	50
Si–(C)(H)_2_(Si)	25	69	34	43	55
Si–(C_D_)(H)_2_(O)	26	53	26	39	51
Si–(F)(H)_2_(O)	–381	159	38	52	68
Si–(C)_3_(H)	–23	–8	32	36	38
Si–(C_D_)_3_(H)	20	–34	21	34	40
Si–(H)(O)_3_	–32	–34	36	39	40
Si–(C)_2_(C_D_)(H)	–9	–16	28	35	39
Si–(C)_2_(H)(O)	–19	–12	32	36	38
Si–(C_D_)_2_(H)(O)	11	–38	25	36	40
Si–(F)_2_(H)(O)	–810	178	47	60	72
Si–(C)(C_D_)_2_(H)	5	–26	26	36	40
Si–(C)(H)(O)_2_	–24	–26	32	37	39
Si–(C_D_)(H)(O)_2_	–7	–35	27	35	39
Si–(F)(H)(O)_2_	–422	71	43	52	58
Si–(C)_4_^a^	–46	–85	35	33	26
Si–(C)_3_(O)^a^	–46	–85	35	33	26
Si–(C)_3_(C_D_)	–32	–87	30	32	26
Si–(C)_3_(Si)	–13	–86	36	35	30
Si–(C)_2_(C_D_)_2_	–19	–106	27	31	27
Si–(C)_2_(O)_2_	–55	–104	35	33	27
Si–(C_D_)_2_(O)_2_	–23	–124	32	39	35
Si–(C)(C_D_)_3_	–6	–116	24	31	28
Si–(C)(O)_3_	–59	–108	36	35	29
Si–(C)(C_D_)(O)_2_	–38	–111	28	31	26
Si–(F)_3_(O)	–1224	214	59	71	78
Si–(F)_2_(O)_2_	–842	87	50	57	60
Si–(O)_4_	–70	–132	43	38	30
C–(C)(H)_2_(Si)	–9	34	22	32	50
C–(C)_2_(H)(Si)	17	–59	19	28	39
O–(H)(Si)	–318	117	14	22	29
O–(C)(Si)	–240	39	5	9	16
O–(Si)_2_	–416	38	10	17	26
ring strain, 6-membered ring	21	87	–5	–3	–3
ring strain, 8-membered ring	4	104	4	5	5

a Values for the group Si–(C)_3_(O) have been fixed to those of Si–(C)_4_.

**Table 4:** Thermochemical Benson Group Pair Contributions for Standard Enthalpies of Formation (Δ_f_*H*_298K_^°^, kJ mol^–1^), Entropies (*S*_298K_^°^, J K^–1^ mol^–1^), and Heat Capacities (*C*_*p*_, J K^–1^ mol^–1^) Derived from the Results of W1X-1 Calculations

Benson group	Δ_f_*H*° 298 K	*S*° 298 K	*C*_*p*_ 298 K	*C*_*p*_ 500 K	*C*_*p*_ 1000 K
Si–(C_B_)(H)_3_ + C_B_–(C_B_)_2_(Si)	56	104	35	58	83
Si–(C)(C_B_)(H)_2_ + C_B_–(C_B_)_2_(Si)	36	37	39	57	74
Si–(C_B_)(H)_2_(O) + C_B_–(C_B_)_2_(Si)	50	31	44	62	80
Si–(C_B_)_2_(H)_2_ + C_B_–(C_B_)_2_(Si)	72	–3	49	74	98
Si–(C)_2_(C_B_)(H) + C_B_–(C_B_)_2_(Si)	13	–47	41	53	62
Si–(C_B_)(C_D_)_2_(H) + C_B_–(C_B_)_2_(Si)	41	–65	36	53	63
Si–(C_B_)(H)(O)_2_ + C_B_–(C_B_)_2_(Si)	14	–63	41	53	62
Si–(C)(C_B_)(C_D_)(H) + C_B_–(C_B_)_2_(Si)	26	–60	37	52	62
Si–(C)(C_B_)(H)(O) + C_B_–(C_B_)_2_(Si)	18	–53	40	53	62
Si–(C)_3_(C_B_) + C_B_–(C_B_)_2_(Si)	–11	–118	44	50	50
Si–(C)_2_(C_B_)_2_ + C_B_–(C_B_)_2_(Si)	22	–167	55	68	73
Si–(C_B_)_2_(O)_2_ + C_B_–(C_B_)_2_(Si)	14	–182	52	66	73
Si–(C)_2_(C_B_)(C_D_) + C_B_–(C_B_)_2_(Si)	2	–135	40	49	50
Si–(C)(C_B_)(C_D_)_2_ + C_B_–(C_B_)_2_(Si)	14	–147	38	50	51
Si–(C)(C_B_)(O)_2_ + C_B_–(C_B_)_2_(Si)	–19	–143	45	52	53
Si–(C_B_)(C_D_)(O)_2_ + C_B_–(C_B_)_2_(Si)	–4	–154	55	70	74
2 Si–(C)_3_(N) + N–(H)(Si)	–218	–134	93	98	96

As discussed earlier, Becerra and Walsh have derived group contributions for silicon-based Benson groups and used them extensively in their work. Comparison of our W1X-1 data in Table 3 with their values shows good agreement with groups Si–(C)(H)_3_, O–(C)(Si), Si–(C)_2_(O)_2_, and Si–(C)(O)_3_ (former values 14, −247, −62, and −61 kJ mol^–1^, respectively).^18^ For all other Benson groups reported by Becerra and Walsh, such as Si–(C)_4_/Si–(C)_3_(O), C–(C)_2_(H)(Si), and O–(Si)_2_, the differences are much greater and even exceed 20 kJ mol^–1^ in some cases. This is entirely expected, considering the large differences seen between W1X-1-calculated enthalpies and the corresponding experimental values.

The group additivity contributions determined herein can also be compared with the work of Janbazi et al.^29,30^ Unfortunately, this is not entirely justified as their data are based on Cohen’s^73^ revised formulation of Benson’s approach.^9^ Furthermore, different values for the groups C–(Si)(H)_3_ and C–(C)(H)_3_ have been chosen by Janbazi et al. to avoid “group-increment analogies”.^29^ Such a choice represents a significant step away from all Benson-type group additivity approaches that uniformly fix the contribution from a methyl group (except for its physical state) no matter what atom it is attached to.^9^ In fact, the work of Janbazi et al. should not be considered an addition to Cohen’s work, but it rather constitutes yet another branch to the ever-growing tree of group additivity approaches.

As discussed earlier, the inaccuracies in the computed enthalpies reported by Janbazi et al.^29,30^ raise concerns over the group contribution values they have determined. In fact, the group contributions given by Janbazi et al. do not reproduce all G4-level enthalpies from which they are derived. For example, differences up to 8 kJ mol^–1^ are found in the methylsilane series, even though the fit to the reference data is claimed to have a maximum deviation of only 0.01 kJ mol^–1^.^29^ More significant is the fact that the values of some group contributions involving oxygen, such as Si–(O)_4_ and Si–(C)(O)_3_, differ considerably, up to 40 kJ mol^–1^, between our data and theirs.^30^ We note that Janbazi et al. do not indicate fixing any of the group contributions involving Si–O bonds. This would allow for an infinite number of equally good fits to their data of which one is presented in the publication. It needs to be stressed that the individual group contributions carry no physical meaning and pre-fixed values, while inherently arbitrary, are important to avoid linear dependencies.

The data reported in Tables 3 and 4 allow for a more accurate estimation of enthalpies of formation for a variety of organosilicon species than has been possible before. In this context, we chose to employ the established values, together with literature values for carbon-based groups,^10,11^ to estimate the standard enthalpies of formation of organosilicon species examined experimentally by Voronkov el al.^20−25^ We have already concluded that their data appear suspicious when compared with the W1X-1 (and W2) enthalpies of formation calculated herein. However, such comparisons could only be made for a handful of compounds as high-level calculations become prohibitively expensive with increasing molecular size. By using group contributions, standard enthalpies of formation can be easily estimated irrespective of molecular size, allowing comparisons not only between bigger systems but also between larger groups of compounds.

Considering tri- and tetrasubstituted alkylsilanes with alkyl chains longer than four carbon atoms, standard enthalpies of formation could be estimated for 22 species examined by Voronkov el al.^20^ The results (Supporting Information) show that the values reported by Voronkov et al. are systematically around 40 kJ mol^–1^ more exothermic than those obtained using group additivity contributions. We feel confident that our values for groups C–(C)(H)_2_(Si), Si–(C)_4_, and Si–(C)_3_(H) are reliable as they reproduce well all W1X-1 enthalpies for tri- and tetrasubstituted alkylsilanes in Table 1. Thus, the data by Voronkov et al. must contain an unknown source of systematic error, as initially suspected by Becerra and Walsh.^17−19^ The published experimental details do not allow us to trace down the origin of the error, but one possible culprit is the standard enthalpy of formation of amorphous hydrated silica whose value is dependent on the exact physical state after combustion. In fact, this problem has been comprehensively studied by Voronkov et al., and the value they use in their work, −939.39 ± 0.52 kJ mol^–1^, stands out from all literature references by being the most exothermic.^74^ Even though an adjustment to this value would make the errors much smaller in the current case, they would, in general, become greater for many other compound classes examined by Voronkov et al. (see below). We therefore conclude that either the exact physical state of amorphous hydrated silica is slightly different for each compound class investigated, which could well be the case, or the experimental data by Voronkov el al. contain more than once source of error.

In the case of longer-chain alkoxysilanes and phenyl-substituted cyclosiloxanes investigated by Voronkov el al.,^20,24^ we found in total 10 compounds for which enthalpies of formation could be estimated using group contributions in Tables 3 and 4 (Supporting Information). For these compounds, the data show no indication of a similar systematic error as seen above, and the differences between the two sets of numbers vary both in sign and in magnitude. However, the absolute differences are smaller for alkoxysilanes than for cyclosiloxanes, and differences much greater than 100 kJ mol^–1^ are seen for cyclosiloxanes with six or eight phenyl groups. It is impossible to assess the origin of this discrepancy with certainty as there are no other experimental data available for comparison and our estimate of the enthalpy contribution associated with the group pair Si–(C_B_)_2_(O)_2_ + C_B_–(C_B_)_2_(Si) is based on a single calculated value due to the computational cost associated with these calculations. We therefore conclude that the experimental data for simple alkoxysilanes published by Voronkov et al.^20^ appear to be of similar quality to many other experimental reports on organosilicon thermochemistry, but there exists a high possibility that the data for cyclosiloxanes are significantly in error.^24^

As a last test, we investigated trimethoxy- and triethoxysilanes with thioether substituents. Voronkov et al. have reported data for 15 compounds of this class,^22^ but only 6 of them can be represented with the Benson groups considered herein and those found in the literature. The results (Supporting Information) are rather remarkable as the differences between experimental and estimated standard enthalpies of formation are less than the associated 3σ confidence intervals in all cases. Consequently, for this particular set of compounds, the data reported by Voronkov et al. are uniformly consistent with our estimations, although the number of compounds to be considered is rather small. It is unfortunately impossible to assess whether the data are inherently better than those of, for example, alkylsilanes or if the better match with our estimates is entirely fortuitous.

## Conclusions

In this work, we established a comprehensive high-accuracy ab initio thermochemical benchmark database for 159 organosilicon compounds using the composite W1X-1 method. The results were compared to W2 level benchmark values and extant experimental data, as well as to prior computational values. The calculated results were also used to derive group additivity contributions for standard gas-phase enthalpy of formation, Δ_f_*H*_298K_^°^, entropy, *S*_298K_^°^, and heat capacity, *C*_*p*_, for 60 Benson groups and group pairs involving silicon that can, in turn, be employed in estimating accurate thermochemical parameters for compounds beyond the limitations imposed by the scaling of the W1X-1 method with respect to molecular size.

The most important results of this work can be summarized as follows:

(i) High-level W1X-1 (and W2) results imply that the experimental standard enthalpies of formation of organosilicon compounds need to be treated with caution, irrespective of their source. As a general trend, when the differences between calculated and experimental enthalpies are observed, experimental values are systematically more exothermic than theoretical predictions. As pointed out in virtually every description of calorimetric analysis of organosilicon compounds, there are numerous possible sources of error in a single experiment and even the most comprehensive studies are not immune to errors that are hard to find and even more difficult to fix. Furthermore, experimental enthalpies of formation can be interdependent, such as those of the methylsilane series, allowing an error in a single value to easily propagate to many others.
(ii) The vast experimental data set of Voronkov et al. is a double-edged sword. On one hand, it contains results, such as the enthalpies of formation of alkylsilanes, which were found to contain a significant systematic error, as initially suspected by Becerra and Walsh. On the other hand, the values reported by Voronkov et al. for alkoxysilanes appear to be no more in error than the results quoted by other authors. The obvious problem is how to differentiate between the two alternatives, and there appears to be no easy answer to this question. Thus, unless the data reported by Voronkov et al. are validated by an independent study, preferably by experimental means, we recommend that they continue to be flagged in thermochemical databases and treated with extreme caution.
(iii) Semi-empirical methods for the estimation of thermochemical properties of molecules are only as accurate as the underlying data used to derive them. The bond and group additivity contributions of Becerra and Walsh are based on experimental data for organosilicon compounds and were found to yield estimates with an accuracy of tens of kJ mol^–1^ at the best. Similarly, inaccuracies in the calculated enthalpies and problems associated with data fitting led to significant differences and incompatibilities between group contributions reported by Janbazi et al. and those from our approach. For these reasons, we consider the W1X-1-based group and group pair contributions reported herein the most accurate and recommend their use in all estimations of thermochemical properties of organosilicon species using Benson’s methodology. In the case of Cohen’s data sets, the values reported herein can be easily converted to comply with the revised parameterization. The W1X-1 data presented in this work also showed that even bond additivity approaches work well for the simplest of cases, for example, for the SiX_*n*_Y_4–*n*_ series, but only if the required substituent replacement enthalpies are determined from accurate enthalpy data.

As a last note, we join Becerra and Walsh and stress the importance of obtaining accurate thermochemical data on chemical compounds and organo-main group species in particular. Since a large-scale renaissance of calorimetry seems unlikely, partly due to limited funding opportunities available for such research, the role played by high-level ab initio theoretical methods, such as W1X-1, in this quest will be crucial. In this respect, we note that the W1X-1 method is currently only able to treat molecules with atoms from the first three rows of the periodic table, that is, up to argon. An extension of this approach to heavier main-group elements, such as germanium and bromine, is a highly desirable objective and currently under development in our group.

## Supporting Information Available

Calculated total energies and energy corrections, symmetry numbers for correction of optical isomerism and internal and external symmetries, and estimated standard enthalpies of formation (PDF)
Optimized molecular coordinates (XYZ)

## Supplementary Material

jp1c09980_si_001.pdf

jp1c09980_si_002.xyz
