# Addition/Correction to “High-Level Ab Initio Predictions of Thermochemical Properties of Organosilicon Species: Critical Evaluation of Experimental Data and a Reliable Benchmark Database for Extending Group Additivity Approaches”

**DOI:** 10.1021/acs.jpca.2c05236

**Published:** 2022-08-17

**Authors:** Hannu T. Vuori, J. Mikko Rautiainen, Erkki T. Kolehmainen, Heikki M. Tuononen

**Affiliations:** Department of Chemistry, Nanoscience Centre, University of Jyväskylä, P.O. Box 35, JyväskyläFI-40014, Finland

Recently, we reported thermochemical properties of a number of organosilicon species calculated at the W1X-1 level of theory.^1^ We have since come to the realization that the atomic reference values used in our original work (Table S1, ESI) were inadvertently based on unrestricted MP2 energies even though the composite W1X-1 protocol uses restricted open-shell wave functions throughout.^2,3^ In this Addition/Correction, we report the revised thermochemical data and demonstrate that they do not change the conclusions of the original paper. The results do, however, provide a cautionary note on the calculation of high-level thermochemical properties for molecules with many heavy (non-hydrogen) atoms.

The performance of restricted open-shell, unrestricted, unrestricted spin contamination corrected, and unrestricted Brueckner doubles variants of the original W1 theory have been discussed in detail by Petersson and co-workers.^4^ The four slightly different methods were found to be virtually indistinguishable on the basis of the data calculated for the G2/97 test set. Though this is certainly true and holds in general for small molecules, even sub-kJ mol^–1^ level systematic variations in atomic reference energies can lead to large differences when the size of the system in question increases considerably. This is clearly shown by our work even though only one of the components of the W1X-1 methodology was based on an unrestricted reference determinant.

Table 1 includes the W1X-1(UMP2) and CBS-QB3 gas phase standard enthalpies of formation published in our original contribution along with the revised W1X-1(ROMP2) values. A comparison of the two W1X-1 data sets shows that the different atomic reference values lead to enthalpies based on restricted open-shell MP2 wave functions being systematically more exothermic (mean absolute difference of 4.2 kJ mol^–1^) compared to the values calculated with the unrestricted formalism. The difference is naturally the smallest for systems with the least number of heavy atoms (e.g., SiH_4_, 0.8 kJ mol^–1^) and grows with respect to the molecular size (e.g., SiPh_2_(OMe)_2_, 8.5 kJ mol^–1^). As a consequence, the revised W1X-1(ROMP2) enthalpies are now in excellent harmony with the CBS-QB3 values for monosilanes **I**, with a positive mean signed deviation (MSD) of only 3 kJ mol^–1^. However, the opposite is true for all other compound classes **II**–**V**, **VI**–**IX**, **X**–**XII**, and **XIII** and **XIV**, and the associated MSD values, −5, −8, −17, and −21 kJ mol^–1^, respectively, are now significantly more negative than those based on the prior W1X-1(UMP2) data.

**Table 1:** Calculated Gas Phase Standard Enthalpies of Formation (Δ_f_*H*°_298K_, kJ mol^–1^) of Monosilanes **1**–**42**, Polysilanes **43**–**49**, Silanols and Alkoxysilanes **50**–**80**, Acyclic Siloxanes **81**–**150**, Cyclic Siloxanes **151**–**158**, and Silylamine **159**^a^

			Δ_f_*H*°_298K_		
group	molecule	chemical formula	CBS-QB3	W1X-1(UMP2)	W1X-1(ROMP2)
**I**	**1**	SiH_4_	27.0	35.9	36.7
	**2**	SiH_3_Me	–27.6	–23.8	–22.5
	**3**	SiH_3_Et	–34.4	–32.8	–31.1
	**4**	SiH_3_Vi	94.3	96.9	98.6
	**5**	SiH_3_Ph	130.6	124.8	131.0
	**6**	SiH_3_^*i*^Pr	–52.7	–52.7	–50.5
	**7**	SiH_3_^*s*^Bu	–70.2	–72.3	–69.6
	**8**	SiH_3_(3-Pe)	–86.8	–90.6	–87.4
	**9**	SiH_2_Me_2_	–85.1	–85.9	–84.1
	**10**	SiH_2_EtMe	–91.9	–94.8	–92.6
	**11**	SiH_2_MeVi	35.9	34.0	36.2
	**12**	SiH_2_MePh	70.5	63.3	67.4
	**13**	SiH_2_Me^*i*^Pr	–110.7	–114.9	–112.2
	**14**	SiH_2_Me^*s*^Bu	–128.0	–134.2	–131.0
	**15**	SiH_2_Me(3-Pe)	–143.8	–151.7	–148.0
	**16**	SiH_2_Et_2_	–98.8	–103.7	–101.0
	**17**	SiH_2_EtPh	62.9	52.7	58.3
	**18**	SiH_2_Vi_2_	156.6	153.5	156.2
	**19**	SiH_2_Ph_2_	223.4	210.3	216.8
	**20**	SiHMe_3_	–145.2	–149.9	–147.7
	**21**	SiHEtMe_2_	–152.1	–158.7	–156.0
	**22**	SiHMe_2_Vi	–24.8	–30.6	–28.0
	**23**	SiHMe_2_Ph	8.5	–2.2	2.4
	**24**	SiHMe_2_^*i*^Pr	–171.0	–178.7	–175.6
	**25**	SiHMe_2_^*s*^Bu	–188.4	–197.9	–194.3
	**26**	SiHMe_2_(3-Pe)	–204.3	–215.3	–211.2
	**27**	SiHEtMePh	–0.8	–12.7	–7.7
	**28**	SiHMeVi_2_	95.1	88.2	91.4
	**29**	SiHMePhVi	127.2	115.7	120.8
	**30**	SiHVi_3_	215.4	207.8	211.5
	**31**	SiHPhVi_2_	247.5	234.9	240.4
	**32**	SiMe_4_	–207.4	–215.0	–212.4
	**33**	SiEtMe_3_	–214.4	–223.7	–220.6
	**34**	SiMe_3_Vi	–87.6	–96.5	–93.3
	**35**	SiMe_3_Ph	–55.2	–68.4	–63.4
	**36**	SiMe_2_Vi_2_	31.8	21.9	25.5
	**37**	SiEtMe_2_Ph	–64.9	–78.8	–73.3
	**38**	SiMe_2_PhVi	63.2	49.1	54.6
	**39**	SiMe_2_Ph_2_	94.0	76.0	83.4
	**40**	SiMeVi_3_	150.1	139.9	143.9
	**41**	SiMePhVi_2_	180.4	166.1	172.1
	**42**	SiEt_4_	–238.8	–251.9	–247.4
**II**	**43**	Si_2_H_6_	74.2	81.1	82.7
	**44**	Si_2_H_5_Me	20.1	22.9	24.9
	**45**	Si_2_H_4_Me_2_	–33.4	–34.6	–32.1
	**46**	Si_2_Me_6_	–267.3	–280.3	–275.9
**III**	**47**	Si_3_H_8_	113.7	120.4	122.7
**IV**	**48**	Si_4_H_10_	151.7	158.4	161.5
**V**	**49**	Si_5_H_12_	189.4	196.1	200.0
**VI**	**50**	SiH_3_OH	–286.7	–280.1	–278.7
	**51**	SiH_2_MeOH	–352.4	–350.7	–348.9
	**52**	SiH_2_EtOH	–359.3	–359.3	–357.0
	**53**	SiHMe_2_OH	–417.7	–419.7	–417.4
	**54**	SiMe_3_OH	–483.4	–488.2	–485.4
	**55**	SiH_3_OMe	–253.7	–245.8	–243.9
	**56**	SiH_2_Me(OMe)	–319.7	–316.4	–314.1
	**57**	SiHMe_2_(OMe)	–384.9	–385.0	–382.3
**VII**	**58**	SiH_2_(OH)_2_	–633.2	–628.7	–626.8
	**59**	SiH_2_(OMe)_2_	–565.7	–557.7	–554.9
	**60**	SiHMe(OMe)_2_	–635.1	–630.3	–627.0
	**61**	SiHVi(OMe)_2_	–513.6	–509.5	–505.7
	**62**	SiHPh(OMe)_2_	–482.7	–483.3	–477.6
	**63**	SiMe_2_(OMe)_2_	–705.1	–702.3	–698.5
	**64**	SiMeVi(OMe)_2_	–584.6	–582.4	–578.1
	**65**	SiMePh(OMe)_2_	–555.0	–556.8	–550.6
	**66**	SiVi_2_(OMe)_2_	–464.3	–462.6	–457.9
	**67**	SiPhVi(OMe)_2_	–435.4	–437.4	–430.7
	**68**	SiPh_2_(OMe)_2_	–406.9	–412.4	–403.9
**VIII**	**69**	SiH(OH)_3_	–988.7	–985.9	–983.5
	**70**	SiMe(OMe)_2_OH	–992.3	–986.6	–982.7
	**71**	SiEt(OMe)_2_OH	–999.1	–994.3	–990.0
	**72**	SiMe(OMe)_3_	–957.0	–948.6	–944.3
	**73**	SiEt(OMe)_3_	–964.3	–956.7	–951.9
**IX**	**74**	Si(OH)_4_	–1344.2	–1341.7	–1338.7
	**75**	Si(OMe)_3_OH	–1243.4	–1232.3	–1227.8
	**76**	Si(OEt)(OMe)_2_OH	–1277.0	–1267.5	–1262.6
	**77**	Si(OEt)_2_(OMe)OH	–1310.8	–1302.8	–1297.4
	**78**	Si(OMe)_4_	–1209.9	–1195.8	–1190.9
	**79**	Si(OEt)(OMe)_3_	–1243.8	–1231.3	–1225.9
	**80**	Si(OEt)_4_	–1345.7	–1337.7	–1330.9
**X**	**81**	O(SiH_3_)_2_	–356.3	–339.7	–337.6
	**82**	O(SiMe_3_)(SiH_3_)	–556.8	–550.7	–547.1
	**83**	O(SiF_3_)(SiH_3_)	–1620.9	–1605.9	–1602.9
	**84**	O(SiH_2_Me)(SiH_3_)	–422.3	–410.7	–408.1
	**85**	O(SiH_2_Vi)(SiH_3_)	–300.1	–289.3	–286.2
	**86**	O(SiH_2_Ph)(SiH_3_)	–265.8	–259.6	–254.7
	**87**	O(SiH_2_F)(SiH_3_)	–774.9	–759.8	–757.3
	**88**	O(SiHMe_2_)(SiH_3_)	–489.3	–481.0	–477.9
	**89**	O(SiHVi_2_)(SiH_3_)	–248.0	–241.3	–237.3
	**90**	O(SiHF_2_)(SiH_3_)	–1204.1	–1190.3	–1187.6
	**91**	O(SiHMePh)(SiH_3_)	–335.7	–332.7	–327.3
	**92**	O(SiH_2_Me)_2_	–488.3	–481.4	–478.3
	**93**	O(SiHMe_2_)(SiH_2_Me)	–555.1	–550.7	–548.0
	**94**	O(SiH_2_Ph)(SiH_2_Me)	–330.6	–329.9	–324.5
	**95**	O(SiMe_3_)(SiH_2_Me)	–622.5	–621.2	–617.2
	**96**	O(SiHMe_2_)_2_	–621.7	–621.6	–617.6
	**97**	O(SiMe_3_)(SiHMe_2_)	–689.1	–690.8	–686.3
	**98**	O(SiMe_3_)_2_	–756.2	–760.0	–755.0
	**99**	O(SiH_2_Vi)_2_	–244.5	–238.9	–234.9
	**100**	O(SiH_2_F)_2_	–1192.1	–1179.1	–1176.4
	**101**	O(SiHF_2_)(SiH_2_F)	–1619.7	–1607.7	–1604.6
	**102**	O(SiF_3_)(SiH_2_F)	–2035.4	–2022.4	–2019.0
	**103**	O(SiHF_2_)_2_	–2045.5	–2034.6	–2031.3
	**104**	O(SiF_3_)(SiHF_2_)	–2460.7	–2448.8	–2445.2
	**105**	O(SiF_3_)_2_	–2874.1	–2861.4	–2857.4
**XI**	**106**	SiH_2_(OSiH_3_)_2_	–771.7	–746.1	–742.7
	**107**	SiH_2_(OSiH_2_Me)(OSiH_3_)	–838.0	–816.3	–812.4
	**108**	SiH_2_(OSiH_2_Vi)(OSiH_3_)	–716.0	–695.9	–691.5
	**109**	SiH_2_(OSiH_2_Ph)(OSiH_3_)	–680.3	–665.7	–659.4
	**110**	SiH_2_(OSiH_2_F)(OSiH_3_)	–1190.8	–1166.0	–1162.2
	**111**	SiH_2_(OSiMe_3_)(OSiH_3_)	–973.7	–958.5	–953.6
	**112**	SiH_2_(OSiHMe_2_)(OSiH_3_)	–905.8	–888.5	–884.1
	**113**	SiH_2_(OSiHF_2_)(OSiH_3_)	–1620.1	–1596.1	–1592.0
	**114**	SiH_2_(OSiF_3_)(OSiH_3_)	–2036.7	–2011.9	–2007.5
	**115**	SiH_2_(OSiH_2_Me)_2_	–904.2	–888.2	–883.8
	**116**	SiH_2_(OSiHMe_2_)(OSiH_2_Me)	–972.0	–959.5	–954.7
	**117**	SiH_2_(OSiMe_3_)(OSiH_2_Me)	–1039.9	–1029.4	–1024.0
	**118**	SiH_2_(OSiH_2_F)_2_	–1607.1	–1585.0	–1581.0
	**119**	SiH_2_(OSiHMe_2_)_2_	–1039.4	–1030.3	–1025.0
	**120**	SiH_2_(OSiMe_3_)(OSiHMe_2_)	–1107.2	–1100.2	–1094.3
	**121**	SiH_2_(OSiMe_3_)_2_	–1175.1	–1169.9	–1163.6
	**122**	SiHMe(OSiH_3_)_2_	–843.2	–821.1	–817.2
	**123**	SiHVi(OSiH_3_)_2_	–720.8	–699.2	–694.8
	**124**	SiHPh(OSiH_3_)_2_	–689.0	–671.9	–665.6
	**125**	SiHF(OSiH_3_)_2_	–1203.6	–1178.3	–1174.5
	**126**	SiHMe(OSiH_2_Me)(OSiH_3_)	–909.5	–892.1	–887.7
	**127**	SiHMe(OSiHMe_2_)(OSiH_3_)	–977.0	–963.4	–958.5
	**128**	SiHMe(OSiMe_3_)(OSiH_3_)	–1045.1	–1033.0	–1027.7
	**129**	SiHMe(OSiH_2_Me)_2_	–975.5	–962.9	–958.0
	**130**	SiHMe(OSiHMe_2_)(OSiH_2_Me)	–1043.4	–1034.4	–1029.0
	**131**	SiHMe(OSiMe_3_)(OSiH_2_Me)	–1111.3	–1104.1	–1098.2
	**132**	SiHMe(OSiHMe_2_)_2_	–1110.6	–1104.4	–1098.6
	**133**	SiHMe(OSiMe_3_)(OSiHMe_2_)	–1179.1	–1174.7	–1168.4
	**134**	SiHMe(OSiMe_3_)_2_	–1246.5	–1243.8	–1237.0
	**135**	SiHF(OSiH_2_F)(OSiH_3_)	–1622.9	–1594.8	–1594.4
	**136**	SiHF(OSiHF_2_)(OSiH_3_)	–2051.1	–2027.1	–2022.7
	**137**	SiMe_2_(OSiH_3_)_2_	–915.5	–894.7	–890.3
	**138**	SiMe_2_(OSiH_2_Me)(OSiH_3_)	–981.5	–965.5	–960.6
	**139**	SiMe_2_(OSiHMe_2_)(OSiH_3_)	–1049.1	–1036.6	–1031.2
	**140**	SiMe_2_(OSiMe_3_)(OSiH_3_)	–1116.9	–1106.2	–1100.4
	**141**	SiMe_2_(OSiH_2_Me)_2_	–1047.4	–1036.0	–1030.6
	**142**	SiMe_2_(OSiHMe_2_)(OSiH_2_Me)	–1115.0	–1107.2	–1101.4
	**143**	SiMe_2_(OSiMe_3_)(OSiH_2_Me)	–1182.8	–1176.7	–1170.4
	**144**	SiMe_2_(OSiMe_3_)_2_	–1317.9	–1316.6	–1309.3
	**145**	SiMe_2_(OSiHMe_2_)_2_	–1182.3	–1177.1	–1170.9
	**146**	SiMe_2_(OSiMe_3_)(OSiHMe_2_)	–1250.4	–1246.9	–1240.1
	**147**	SiF_2_(OSiH_3_)_2_	–1625.4	–1598.9	–1594.8
	**148**	SiF_2_(OSiH_2_F)(OSiH_3_)	–2043.8	–2017.1	–2012.7
	**149**	SiF_2_(OSiH_2_F)_2_	–2460.5	–2434.9	–2430.3
**XII**	**150**	O(SiH_2_OSiH_3_)_2_	–1186.1	–1151.9	–1147.1
**XIII**	**151**	(OSiH_2_)_3_	–1215.7	–1196.3	–1192.4
	**152**	(OSiHMe)(OSiH_2_)_2_	–1290.0	–1273.4	–1269.0
	**153**	(OSiMe_2_)(OSiH_2_)_2_	–1362.9	–1348.1	–1343.2
	**154**	(OSiHMe)_2_(OSiH_2_)	–1363.8	–1350.2	–1345.3
	**155**	(OSiMe_2_)(OSiHMe)(OSiH_2_)	–1436.6	–1424.6	–1419.1
	**156**	(OSiHMe)_3_	–1437.3	–1426.6	–1421.2
	**157**	(OSiMe_2_)_3_	–1653.8	–1648.3	–1641.5
**XIV**	**158**	(OSiH_2_)_4_	–1656.2	–1623.5	–1618.1
**XV**	**159**	NH(SiMe_3_)_2_	–454.0	–472.0	–457.5

a Used abbreviations: Me = methyl, Et = ethyl, ^*i*^Pr = isopropyl, ^*s*^Bu = *sec*-butyl, 3-Pe = 3-pentyl, Vi = vinyl, and Ph = phenyl.

Table 2 lists well-established experimental gas phase standard enthalpies of formation for 13 reference silicon compounds used in our original paper along with the calculated values. Unsurprisingly, the W1X-1(UMP2) and W1X-1(ROMP2) values are nearly identical for the structurally simplest alkylsilanes and silanols, with larger differences observed for systems with multiple methyl and ethyl substituents, such as Si_2_Me_6_ and Si(OEt)_4_, in which case the W1X-1(ROMP2) and W2 data are in good agreement with each other. The conclusions in our original paper are unaffected by the changes in the calculated values, and we continue to stress the importance of obtaining accurate experimental thermochemical data on compounds such as SiMe_4_ and Si(OEt)_4_ for which large differences between reference values and W2 level calculations are observed.

**Table 2:** Experimental (exptl) and Calculated (CBS-QB3, W1X-1(UMP2), W1X-1(ROMP2), and W2) Gas Phase Standard Enthalpies of Formation (Δ_f_*H*°_298K_, kJ mol^–1^) of 13 Reference Silicon Compounds^a^

	Δ_f_*H*°_298K_				
molecule	exptl	CBS-QB3	W1X-1(UMP2)	W1X-1(ROMP2)	W2
SiH_4_	34.3 ± 1.2	27.0	35.9	36.7	
Si_2_H_6_	79.9 ± 1.5	74.2	81.1	82.7	
Si_3_H_8_	120.9 ± 4.4	113.7	120.4	122.7	
SiH_3_Me	–29.1 ± 4.0	–27.6	–23.8	–22.5	
SiH_2_Me_2_	–94.7 ± 4.0	–85.1	–85.9	–84.1	
SiHMe_3_	–163.4 ± 4.0	–145.2	–149.9	–147.7	
SiMe_4_	–233.2 ± 3.2	–207.4	–215.0	–212.4	–212.8
Si_2_Me_6_	–303.7 ± 5.5	–267.3	–280.3	–275.9	–277.0
Si(OH)_4_	–1351.3 ± 1.7	–1344.2	–1341.7	–1338.7	
SiMe_3_(OH)	–500.0 ± 3.0	–483.4	–488.2	–485.4	
Si(OEt)_4_	–1356.0 ± 6.0	–1345.7	–1337.7	–1330.9	–1331.4
O(SiMe_3_)_2_	–777.4 ± 6.0	–756.2	–760.0	–755.0	–761.0
NH(SiMe_3_)_2_	–477.0 ± 5.0	–454.0	–472.0	–457.5	–460.8

a Experimental data are taken from the two most recent compilations by Becerra and Walsh.^5,6^

In our original paper, we noted significant differences between our W1X-1(UMP2) values for the methylsilane series and the G4 enthalpies reported by Janbazi et al.,^7,8^+26.2, −87.3, −160.0, and −233.6 kJ mol^–1^, for SiH_3_Me, SiH_2_Me_2_, SiHMe_3_, and SiMe_4_, respectively. These differences persist even after our data have been adjusted to use atomic energies based on restricted open-shell MP2 wave functions (see Table 2). Considering the identical values given by W1X-1(ROMP2) and W2 for the standard enthalpy of formation of SiMe_4_, −212.4 and −212.8 kJ mol^–1^, respectively, the G4 prediction by Janbazi et al. remains questionable even though it matches the well-established experimental value, −233.2 ± 3.2 kJ mol^–1^.

The last effort reported in our original contribution focused on using the calculated W1X-1 thermochemical data to derive Benson group contributions for 60 silicon-based groups and group pairs. The revised group values based on the W1X-1(ROMP2) energies are given in Tables 3 and 4. We note that the values in Table 3 are nearly, within a few kJ mol^–1^, identical with the original data. In line with the discussion in the original paper, these values can be considered superior over those reported by Janbazi et al. and Becerra and Walsh. The values for group pairs given in Table 4 show, however, greater variation with respect to the original W1X-1(UMP2) data. This is to be expected because they are derived from enthalpies calculated for bigger molecules with aromatic substituents. Overall, the revised values reported herein are the most accurate ones determined to date and we recommend their use in all estimations of thermochemical properties of organosilicon species using Benson’s methodology.

**Table 3:** Thermochemical Benson Group Contributions for Standard Enthalpies of Formation (Δ_f_*H*°_298K_, kJ mol^–1^), Entropies (*S*°_298K_, J K^–1^ mol^–1^), and Heat Capacities (*C*_*p*_, J K^–1^ mol^–1^) Derived from Results of W1X-1(ROMP2) calculations

Benson group	Δ_f_*H*°_298K_	*S*°_298K_	*C*_*p* 298K_	*C*_*p* 500K_	*C*_*p* 1000K_		Benson group	Δ_f_*H*°_298K_	*S*°_298K_	*C*_*p* 298K_	*C*_*p* 500K_	*C*_*p* 1000K_
Si–(C)(H)_3_	20	156	32	45	63		Si–(C_D_)(H)(O)_2_	–4	–35	27	35	39
Si–(C_D_)(H)_3_	36	149	28	45	64		Si–(F)(H)(O)_2_	–421	71	43	52	58
Si–(H)_3_(O)	39	151	30	44	63		Si–(C)_4_^a^	–43	–85	35	33	26
Si–(H)_3_(Si)	41	152	35	49	68		Si–(C)_3_(O)^a^	–43	–85	35	33	26
Si–(C)_2_(H)_2_	0	72	31	40	51		Si–(C)_3_(C_D_)	–29	–87	30	32	26
Si–(C_D_)_2_(H)_2_	31	53	25	40	52		Si–(C)_3_(Si)	–11	–86	36	35	30
Si–(H)_2_(O)_2_	11	56	31	41	51		Si–(C)_2_(C_D_)_2_	–15	–106	27	31	27
Si–(H)_2_(Si)_2_	39	68	36	46	59		Si–(C)_2_(O)_2_	–53	–104	35	33	27
Si–(C)(C_D_)(H)_2_	16	63	28	40	51		Si–(C_D_)_2_(O)_2_	–19	–124	32	39	35
Si–(C)(H)_2_(O)	11	63	30	39	50		Si–(C)(C_D_)_3_	–2	–116	24	31	28
Si–(C)(H)_2_(Si)	26	69	34	43	55		Si–(C)(O)_3_	–57	–108	36	35	29
Si–(C_D_)(H)_2_(O)	28	53	26	39	51		Si–(C)(C_D_)(O)_2_	–35	–111	28	31	26
Si–(F)(H)_2_(O)	–380	159	38	52	68		Si–(F)_3_(O)	–1222	214	59	71	78
Si–(C)_3_(H)	–20	–8	32	36	38		Si–(F)_2_(O)_2_	–840	87	50	57	60
Si–(C_D_)_3_(H)	24	–34	21	34	40		Si–(O)_4_	–67	–132	43	38	30
Si–(H)(O)_3_	–30	–34	36	39	40		C–(C)(H)_2_(Si)	–9	34	22	32	50
Si–(C)_2_(C_D_)(H)	–6	–16	28	35	39		C–(C)_2_(H)(Si)	18	–59	19	28	39
Si–(C)_2_(H)(O)	–16	–12	32	36	38		O–(H)(Si)	–318	117	14	22	29
Si–(C_D_)_2_(H)(O)	14	–38	25	36	40		O–(C)(Si)	–240	39	5	9	16
Si–(F)_2_(H)(O)	–808	178	47	60	72		O–(Si)_2_	–416	38	10	17	26
Si–(C)(C_D_)_2_(H)	8	–26	26	36	40		ring strain, 6-membered ring	21	87	–5	–3	–3
Si–(C)(H)(O)_2_	–22	–26	32	37	39		ring strain, 8-membered ring	4	104	4	5	5

a Values for the group Si–(C)_3_(O) have been fixed to those of Si–(C)_4_.

**Table 4:** Thermochemical Benson Group Pair Contributions for Standard Enthalpies of Formation (Δ_f_*H*°_298K_, kJ mol^–1^), Entropies (*S*°_298K_, J K^–1^ mol^–1^), and Heat Capacities (*C*_*p*_, J K^–1^ mol^–1^) Derived from Results of W1X-1 Calculations

Benson group	Δ_f_*H*°_298K_	*S*°_298K_	*C*_*p* 298K_	*C*_*p* 500K_	*C*_*p* 1000K_
Si–(C_B_)(H)_3_ + C_B_–(C_B_)_2_(Si)	62	104	35	58	83
Si–(C)(C_B_)(H)_2_ + C_B_–(C_B_)_2_(Si)	40	37	39	57	74
Si–(C_B_)(H)_2_(O) + C_B_–(C_B_)_2_(Si)	54	31	44	62	80
Si–(C_B_)_2_(H)_2_ + C_B_–(C_B_)_2_(Si)	79	–3	49	74	98
Si–(C)_2_(C_B_)(H) + C_B_–(C_B_)_2_(Si)	17	–47	41	53	62
Si–(C_B_)(C_D_)_2_(H) + C_B_–(C_B_)_2_(Si)	46	–65	36	53	63
Si–(C_B_)(H)(O)_2_ + C_B_–(C_B_)_2_(Si)	18	–63	41	53	62
Si–(C)(C_B_)(C_D_)(H) + C_B_–(C_B_)_2_(Si)	31	–60	37	52	62
Si–(C)(C_B_)(H)(O) + C_B_–(C_B_)_2_(Si)	23	–53	40	53	62
Si–(C)_3_(C_B_) + C_B_–(C_B_)_2_(Si)	–6	–118	44	50	50
Si–(C)_2_(C_B_)_2_ + C_B_–(C_B_)_2_(Si)	30	–167	55	68	73
Si–(C_B_)_2_(O)_2_ + C_B_–(C_B_)_2_(Si)	22	–182	52	66	73
Si–(C)_2_(C_B_)(C_D_) + C_B_–(C_B_)_2_(Si)	7	–135	40	49	50
Si–(C)(C_B_)(C_D_)_2_ + C_B_–(C_B_)_2_(Si)	20	–147	38	50	51
Si–(C)(C_B_)(O)_2_ + C_B_–(C_B_)_2_(Si)	–14	–143	45	52	53
Si–(C_B_)(C_D_)(O)_2_ + C_B_–(C_B_)_2_(Si)	1	–154	55	70	74
2*Si–(C)_3_(N) + N–(H)(Si)_2_	–204	–134	93	98	96

The data reported in Tables 3 and 4 were used to estimate the standard enthalpies of formation of organosilicon species examined experimentally by Voronkov et al.^9−14^ An updated version of Table S4 published in the Supporting Information of our original paper is presented herein as Table 5. The reported values reproduce the same trends as discussed earlier: a systematic difference of around 40 kJ mol^–1^ is seen in the data for tri- and tetrasubstituted alkylsilanes, wildly varying data are observed for longer-chain alkoxysilanes and phenyl-substituted cyclosiloxanes, and excellent harmony between experimental and estimated enthalpies of formation is noted for trimethoxy- and triethoxysilanes with thioether substituents. Thus, we reiterate our earlier conclusion that the data reported by Voronkov et al. should be flagged in thermochemical databases and treated with caution.

**Table 5:** Comparison between Experimental (exptl) and Estimated (Benson) Standard Gas Phase Enthalpies of Formation (Δ_f_*H*°_298K_, kJ mol^–1^) of Organosilicon Compounds Studied by Voronkov et al.^a^

chemical formula	Benson groups^b^^,^^c^	Δ_f_*H*°_298K_ exptl	Δ_f_*H*°_298K_ Benson	diff
SiH(C_4_H_9_)_3_	6*C–(C)_2_(H)_2_, 3*C–(C)(H)_3_, 3*C–(C)(H)_2_*(Si), Si–(H)(C)*_3_	–341.0	–298	**43**
SiH(C_5_H_11_)_3_	9*C–(C)_2_(H)_2_, 3*C–(C)(H)_3_, 3*C–(C)(H)_2_*(Si), Si–(H)(C)*_3_	–402.0	–359	**43**
SiH(C_6_H_13_)_3_	12*C–(C)_2_(H)_2_, 3*C–(C)(H)_3_, 3*C–(C)(H)_2_*(Si), Si–(H)(C)*_3_	–466.0	–421	**45**
SiH(C_7_H_15_)_3_	15*C–(C)_2_(H)_2_, 3*C–(C)(H)_3_, 3*C–(C)(H)_2_*(Si), Si–(H)(C)*_3_	–529.0	–483	**46**
SiH(C_8_H_17_)_3_	18*C–(C)_2_(H)_2_, 3*C–(C)(H)_3_, 3*C–(C)(H)_2_*(Si), Si–(H)(C)*_3_	–591.0	–545	**46**
SiH(C_9_H_19_)_3_	21*C–(C)_2_(H)_2_, 3*C–(C)(H)_3_, 3*C–(C)(H)_2_*(Si), Si–(H)(C)*_3_	–651.0	–607	**44**
SiH(C_10_H_21_)_3_	24*C–(C)_2_(H)_2_, 3*C–(C)(H)_3_, 3*C–(C)(H)_2_*(Si), Si–(H)(C)*_3_	–713.0	–669	**44**
SiH(*s*-C_4_H_9_)_3_	6*C–(C)(H)_3_, 3*C–(C)_3_(H), 3*tert., 3*C–(C)(H)_2_*(Si), Si–(H)(C)*_3_	–355.0	–311	**44**
SiH(*i*-C_5_H_11_)_3_	6*C–(C)(H)_3_, 3*C–(C)_2_(H)_2,_ 3*C–(C)_3_(H), 3*tert., 3*C–(C)(H)_2_*(Si), Si–(H)(C)*_3_	–413.0	–373	**40**
SiH(CH_3_)(C_4_H_9_)_2_	4*C–(C)_2_(H)_2_, 2*C–(C)(H)_3_, C–(H)_3_(Si), 2*C–(C)(H)_2_*(Si), Si–(H)(C)*_3_	–283.0	–247	**36**
SiH(CH_3_)(C_5_H_11_)_2_	6*C–(C)_2_(H)_2_, 2*C–(C)(H)_3_, C–(H)_3_(Si), 2*C–(C)(H)_2_*(Si), Si–(H)(C)*_3_	–325.0	–289	**36**
SiH(CH_3_)(C_6_H_13_)_2_	8*C–(C)_2_(H)_2_, 2*C–(C)(H)_3_, C–(H)_3_(Si), 2*C–(C)(H)_2_*(Si), Si–(H)(C)*_3_	–366.0	–330	**36**
SiH(CH_3_)(C_10_H_21_)_2_	16*C–(C)_2_(H)_2_, 2*C–(C)(H)_3_, C–(H)_3_(Si), 2*C–(C)(H)_2_*(Si), Si–(H)(C)*_3_	–531.0	–495	**36**
SiH(C_2_H_5_)(C_4_H_9_)_2_	4*C–(C)_2_(H)_2_, 3*C–(C)(H)_3_, 3*C–(C)(H)_2_*(Si), Si–(H)(C)*_3_	–301.0	–256	**45**
SiH(C_2_H_5_)(C_5_H_11_)_2_	6*C–(C)_2_(H)_2_, 3*C–(C)(H)_3_, 3*C–(C)(H)_2_*(Si), Si–(H)(C)*_3_	–340.0	–298	**42**
SiH(C_2_H_5_)(C_6_H_13_)_2_	8*C–(C)_2_(H)_2_, 3*C–(C)(H)_3_, 3*C–(C)(H)_2_*(Si), Si–(H)(C)*_3_	–381.0	–339	**42**
SiH(C_2_H_5_)(C_8_H_17_)_2_	12*C–(C)_2_(H)_2_, 3*C–(C)(H)_3_, 3*C–(C)(H)_2_*(Si), Si–(H)(C)*_3_	–468.0	–421	**47**
SiH(C_2_H_5_)(C_10_H_21_)_2_	16*C–(C)_2_(H)_2_, 3*C–(C)(H)_3_, 3*C–(C)(H)_2_*(Si), Si–(H)(C)*_3_	–545.0	–504	**41**
SiH(C_2_H_5_)(*s*-C_4_H_9_)_2_	5*C–(C)(H)_3_, 2*C–(C)_3_(H), 2*tert., 3*C–(C)(H)_2_*(Si), Si–(H)(C)*_3_	–315.0	–265	**50**
SiH(C_2_H_5_)(*i*-C_5_H_11_)_2_	5*C–(C)(H)_3_, 2*C–(C)_2_(H)_2_, 2*C–(C)_3_(H), 2*tert., 3*C–(C)(H)_2_*(Si), Si–(H)(C)*_3_	–358.0	–306	**52**
Si(C_3_H_7_)_2_(C_4_H_9_)_2_	6*C–(C)_2_(H)_2_, 4*C–(C)(H)_3_, 4*C–(C)(H)_2_*(Si), Si–(C)*_4_	–423.0	–372	**51**
Si(C_3_H_7_)(C_4_H_9_)_3_	7*C–(C)_2_(H)_2_, 4*C–(C)(H)_3_, 4*C–(C)(H)_2_*(Si), Si–(C)*_4_	–444.0	–392	**52**
Si(C_3_H_7_)_2_(OC_2_H_5_)_2_	4*C–(C)(H)_3_, 2*C–(C)_2_(H)_2_, 2*C–(C)(H)_2_(O), 2*C–(C)(H)_2_(Si), 2*O–(C)(Si), Si–(C)_2_*(O)*_2_	–852.0	–827	**25**
Si(OC_3_H_7_)_4_	4*C–(C)(H)_3_, 4*C–(C)_2_(H)_2_, 4*C–(C)(H)_2_(O), 4*O–(C)(Si), Si–(O)_4_	–1397.0	–1410	**–13**
Si(OC_4_H_9_)_4_	8*C–(C)_2_(H)_2_, 4*C–(C)(H)_3_, 4*C–(C)(H)_2_(O), 4*O–(C)(Si), Si–(O)_4_	–1482.0	–1493	**–11**
(OSiPh_2_)_3_	30*C_B_–(C_B_)_2_(H), 3*[Si–(C_*B*_*)*_2_*(O)*_2_*+ C*_*B*_*–(C*_*B*_*)*_2_(Si)], 3*O–(Si)_2_, *6-member*	–880.0	–747	**133**
(OSiMe_2_)_4_	8*C–(H)_3_(Si), 4*Si–(C)_2_*(O)*_2_, 4*O–(Si)_2_, *8-member*	–2138.0	–2210	**–72**
(OSiMe_2_)(OSiPh_2_)_3_	30*C_B_–(C_B_)_2_(H), 2*C–(H)_3_(Si), 4*O–(Si)_2_, 3*[Si–(C_*B*_*)*_2_*(O)*_2_*+ C*_*B*_*–(C*_*B*_*)*_2_*(Si)], Si–(C)*_2_*(O)*_2_, *8-member*	–1454.0	–1317	**137**
(OSiMe_2_)_2_(OSiPh_2_)_2_	20*C_B_–(C_B_)_2_(H), 4*C–(H)_3_(Si), 4*O–(Si)_2_, 2*Si–(C)_2_*(O)*_2_, 2*[Si–(C_*B*_*)*_2_*(O)*_2_*+ C*_*B*_*–(C*_*B*_*)*_2_*(Si)], 8-member*	–1691.0	–1615	**76**
(OSiMe_2_)_3_(OSiPh_2_)	10*C_B_–(C_B_)_2_(H), 6*C–(H)_3_(Si), 4*O–(Si)_2_, 3*Si–(C)_2_*(O)*_2_, *[Si–(C*_*B*_*)*_2_*(O)*_2_*+ C*_*B*_*–(C*_*B*_*)*_2_*(Si)], 8-member*	–1910.0	–1912	**–2**
(OSiPh_2_)_4_	40*C_B_–(C_B_)_2_(H), 4*[Si–(C_*B*_*)*_2_*(O)*_2_*+ C*_*B*_*–(C*_*B*_*)*_2_(Si)], 4*O–(Si)_2_, *8-member*	–1226.0	–1020	**206**
(OSiMePh)_4_	4*C–(H)_3_(Si), 20*C_B_–(C_B_)_2_(H), 4*[Si–(C)(C_*B*_*)(O)*_2_*+ C*_*B*_*–(C*_*B*_*)*_2_(Si)], 4*O–(Si)_2_, *8-member*	–1685.0	–1609	**76**
Si(OCH_3_)_3_[(CH_2_)_2_SCH_3_]	3*C–(H)_3_(O), C–(C)(H)_2_(S), C–(H)_3_(S), S–(C)_2_, 3*O–(C)(Si), C–(C)(H)_2_*(Si), Si–(C)(O)*_3_	–946.6	–933	**15**
Si(OCH_3_)_3_[(CH_2_)_3_SCH_3_]	3*C–(H)_3_(O), C–(C)_2_(H)_2_, C–(C)(H)_2_(S), C–(H)_3_(S), S–(C)_2_, 3*O–(C)(Si), C–(C)(H)_2_*(Si), Si–(C)(O)*_3_	–957.0	–954	**5**
Si(OCH_3_)_3_[(CH_2_)_2_S(CH_2_CH_3_)]	3*C–(H)_3_(O), 2*C–(C)(H)_2_(S), C–(H)_3_(C), S–(C)_2_, 3*O–(C)(Si), C–(C)(H)_2_*(Si), Si–(C)(O)*_3_	–962.2	–956	**8**
Si(OCH_3_)_3_[(CH_2_)_3_S(CH_2_CH_3_)]	3*C–(H)_3_(O), 2*C–(C)(H)_2_(S), C–(C)_2_(H)_2_, C–(H)_3_(C), S–(C)_2_, 3*O–(C)(Si), C–(C)(H)_2_*(Si), Si–(C)(O)*_3_	–979.9	–977	**5**
Si(OCH_2_CH_3_)_3_[(CH_2_)_2_S(CH_2_CH_3_)]	4*C–(C)(H)_3_, 3*C–(C)(H)_2_(O), 2*C–(C)(H)_2_(S), S–(C)_2_, 3*O–(C)(Si), C–(C)(H)_2_*(Si), Si–(C)(O)*_3_	–1069.0	–1055	**16**
Si(OCH_2_CH_3_)_3_[(CH_2_)_3_S(CH_2_)_3_CH_3_]	4*C–(C)(H)_3_, 3*C–(C)_2_(H)_2_, 3*C–(C)(H)_2_(O), 2*C–(C)(H)_2_(S), S–(C)_2_, 3*O–(C)(Si), C–(C)(H)_2_*(Si), Si–(C)(O)*_3_	–1119.0	–1115	**4**

a See refs (9−14) for details of the experimental work.

b Literature values (kJ mol^–1^): C–(C)(H)_3_ = C–(H)_3_(O) = C–(H)_3_(Si) = −42.26, C–(C)_4_ = 19.2, C–(C)_3_(H) = −1.17, C–(C)_2_(H)_2_ = −20.63, C_B_–(C_B_)_2_(H) = −13.81, C–(C)(H)_2_(O) = −32.90, C–(C)(H)_2_(S) = −23.17, S–(C)_2_ = 46.99, tertiary corr = −2.26.

c Determined in this work (*italicized*, kJ mol^–1^): C–(C)_2_(H)(Si) = 18, C–(C)(H)_2_(Si) = −9, Si–(C)_4_ = −43, Si–(C)_3_(H) = −20, Si–(C)_2_(O)_2_ = −53, [Si–(C)(C_B_)(O)_2_ + C_B_–(C_B_)_2_(Si)] = −14, [Si–(C_B_)_2_(O)_2_ + C_B_–(C_B_)_2_(Si)] = 22, Si–(C)(O)_3_ = −57, Si–(C)(H)(O)_2_ = −22, Si–(O)_4_ = −67, O–(C)(Si) = −240, O–(Si)_2_ = −416, 6-member ring corr = 21, 8-member ring corr = 4.

As a last note, while comparing our atomic reference energies to those reported in the original W1X-1 work, we noticed that the protocol for the determination of extrapolation exponents α for ΔCCSD and Δ(T) energy components had not been described in detail.^2^ A later publication by the same author, however, confirmed that the exponents were determined simultaneously by fitting the energies to the G2/97 set of thermochemical quantities.^15^ Although this leads to excellent performance based on the reported benchmark data, it does allow the ΔCCSD and Δ(T) energy components to compensate for one another in a manner that might not work equally well for all possible molecular systems. Consequently, it is entirely possible that the differences between W1X-1 and CBS-QB3 values noted by us (see above) are not entirely due to deficiencies in the latter method but can also be affected by the extrapolation exponents α used in the former. More detailed investigations of the performance of W1X-1 method with respect to the original W1 and W2 variants are currently underway.
