# Supplementary material for: High-Level Ab Initio Predictions of Thermochemical Properties of Organosilicon Species: Critical Evaluation of Experimental Data and a Reliable Benchmark Database for Extending Group Additivity Approaches
Source: J Phys Chem A. 2022 Mar 7;126(10):1729–42. doi: 10.1021/acs.jpca.1c09980 (PMC8935367; doi:10.1021/acs.jpca.1c09980)
Supplement: Supplementary file 1 — jp1c09980_si_001.pdf [file jp1c09980_si_001.pdf]

Supporting information for

High-Level *ab Initio* Predictions of Thermochemical Properties of Organosilicon Species: Critical Evaluation of Experimental Data and a Reliable Benchmark Database for Extending Group Additivity Approaches

Hannu T. Vuori, J. Mikko Rautiainen, Erkki T. Kolehmainen, and Heikki M. Tuononen**

Department of Chemistry, Nanoscience Centre, P.O. Box 35, University of Jyväskylä, FI-40014, Finland.

Table S1. Standard enthalpies of formation ($\Delta_f H°$, 298 K, kJ mol^{-1}) and calculated total energies (E_{tot}, 0 K, a.u.) of gaseous atoms in their ground state.

Element	$\Delta_f H°_{gas}$ 298 K	CBS-QB3 E_{tot} 0 K	W1X-1 E_{tot} 0 K
H	217.999	-0.499973	-0.497457
C	716.670	-37.849910	-37.783017
N	472.683	-54.603358	-54.518176
O	249.173	-75.105636	-74.985269
F	79.390	-99.804069	-99.640698
Si	452.709	-289.795504	-288.928940

Table S2. Point groups and total, internal, and external symmetry numbers (σ_{tot}, σ_{int}, and σ_{ext}) of compounds **1**–**159**.

Group	#	Chemical Formula	Point Group	σ_{tot}	σ_{int}	σ_{ext}
**I**	**1**	SiH_4	T_d	12	1	12
	**2**	SiH_3Me	C_{3v}	9	3	3
	**3**	SiH_3Et	C_s	9	9	1
	**4**	SiH_3Vi	C_s	3	3	1
	**5**	SiH_3Ph	C_s	6	6	1
	**6**	$SiH_3{}^iPr$	C_s	27	27	1
	**7**	$SiH_3{}^sBu$	C_1	27	27	1
	**8**	SiH_3(3-Pe)	C_1	27	27	1
	**9**	SiH_2Me_2	C_{2v}	18	9	2
	**10**	SiH_2EtMe	C_s	9	9	1
	**11**	SiH_2MeVi	C_1	3	3	1
	**12**	SiH_2MePh	C_s	6	6	1
	**13**	$SiH_2Me{}^iPr$	C_1	27	27	1
	**14**	$SiH_2Me{}^sBu$	C_1	27	27	1
	**15**	SiH_2Me(3-Pe)	C_1	27	27	1
	**16**	SiH_2Et_2	C_{2v}	18	9	2
	**17**	SiH_2EtPh	C_s	6	6	1
	**18**	SiH_2Vi_2	C_2	2	1	2
	**19**	SiH_2Ph_2	C_2	8	4	2
	**20**	$SiHMe_3$	C_{3v}	81	27	3
	**21**	$SiHEtMe_2$	C_1	27	27	1
	**22**	$SiHMe_2Vi$	C_s	9	9	1
	**23**	$SiHMe_2Ph$	C_s	18	18	1
	**24**	$SiHMe_2{}^iPr$	C_s	81	81	1
	**25**	$SiHMe_2{}^sBu$	C_1	81	81	1
	**26**	$SiHMe_2$(3-Pe)	C_1	81	81	1
	**27**	$SiHEtMePh$	C_1	18	18	1
	**28**	$SiHMeVi_2$	C_1	3	3	1
	**29**	$SiHMePhVi$	C_1	6	6	1
	**30**	$SiHVi_3$	C_1	1	1	1
	**31**	$SiHPhVi_2$	C_s	2	2	1
	**32**	$SiMe_4$	T_d	972	81	12
	**33**	$SiEtMe_3$	C_s	243	243	1
	**34**	$SiMe_3Vi$	C_s	81	81	1
	**35**	$SiMe_3Ph$	C_s	162	162	1
	**36**	$SiMe_2Vi_2$	C_2	18	9	2
	**37**	$SiEtMe_2Ph$	C_1	54	54	1
	**38**	$SiMe_2PhVi$	C_1	18	18	1
	**39**	$SiMe_2Ph_2$	C_2	72	36	2

Table S2. Point groups and total, internal, and external symmetry numbers (σ_{tot}, σ_{int}, and σ_{ext}) of compounds **1–159**.

Group	#	Chemical Formula	Point Group	σ_{tot}	σ_{int}	σ_{ext}
	**40**	$SiMeVi_3$	C_3	9	3	3
	**41**	$SiMePhVi_2$	C_1	6	6	1
	**42**	$SiEt_4$	D_{2d}	324	81	4
**II**	**43**	Si_2H_6	D_{3d}	18	3	6
	**44**	Si_2H_5Me	C_s	9	9	1
	**45**	$Si_2H_4Me_2$	C_{2h}	18	9	2
	**46**	Si_2Me_6	D_{3d}	13122	2187	6
**III**	**47**	Si_3H_8	C_{2v}	18	9	2
**IV**	**48**	Si_4H_{10}	C_{2h}	18	9	2
**V**	**49**	Si_5H_{12}	C_{2v}	18	9	2
**VI**	**50**	SiH_3OH	C_s	3	3	1
	**51**	SiH_2MeOH	C_s	3	3	1
	**52**	SiH_2EtOH	C_1	3	3	1
	**53**	$SiHMe_2OH$	C_1	9	9	1
	**54**	$SiMe_3OH$	C_s	81	81	1
	**55**	SiH_3OMe	C_s	9	9	1
	**56**	$SiH_2Me(OMe)$	C_s	9	9	1
	**57**	$SiHMe_2(OMe)$	C_1	27	27	1
**VII**	**58**	$SiH_2(OH)_2$	C_2	2	1	2
	**59**	$SiH_2(OMe)_2$	C_2	18	9	2
	**60**	$SiHMe(OMe)_2$	C_1	27	27	1
	**61**	$SiHVi(OMe)_2$	C_1	9	9	1
	**62**	$SiHPh(OMe)_2$	C_1	18	18	1
	**63**	$SiMe_2(OMe)_2$	C_2	162	81	2
	**64**	$SiMeVi(OMe)_2$	C_1	27	27	1
	**65**	$SiMePh(OMe)_2$	C_1	54	54	1
	**66**	$SiVi_2(OMe)_2$	C_2	18	9	2
	**67**	$SiPhVi(OMe)_2$	C_1	18	18	1
	**68**	$SiPh_2(OMe)_2$	C_2	72	36	2
**VIII**	**69**	$SiH(OH)_3$	C_1	1	1	1
	**70**	$SiMe(OMe)_2OH$	C_1	27	27	1
	**71**	$SiEt(OMe)_2OH$	C_1	27	27	1
	**72**	$SiMe(OMe)_3$	C_1	81	81	1
	**73**	$SiEt(OMe)_3$	C_1	81	81	1
**IX**	**74**	$Si(OH)_4$	S_4	2	1	2
	**75**	$Si(OMe)_3OH$	C_1	27	27	1
	**76**	$Si(OEt)(OMe)_2OH$	C_1	27	27	1
	**77**	$Si(OEt)_2(OMe)OH$	C_1	27	27	1
	**78**	$Si(OMe)_4$	S_4	162	81	2

Table S2. Point groups and total, internal, and external symmetry numbers (σ_{tot}, σ_{int}, and σ_{ext}) of compounds **1–159**.

Group	#	Chemical Formula	Point Group	σ_{tot}	σ_{int}	σ_{ext}
	**79**	$Si(OEt)(OMe)_3$	C_1	81	81	1
	**80**	$Si(OEt)_4$	S_4	162	81	2
**X**	**81**	$O(SiH_3)_2$	C_{2v}	18	9	2
	**82**	$O(SiMe_3)(SiH_3)$	C_s	243	243	1
	**83**	$O(SiF_3)(SiH_3)$	C_1	9	9	1
	**84**	$O(SiH_2Me)(SiH_3)$	C_s	9	9	1
	**85**	$O(SiH_2Vi)(SiH_3)$	C_1	3	3	1
	**86**	$O(SiH_2Ph)(SiH_3)$	C_1	6	6	1
	**87**	$O(SiH_2F)(SiH_3)$	C_1	3	3	1
	**88**	$O(SiHMe_2)(SiH_3)$	C_s	27	27	1
	**89**	$O(SiHVi_2)(SiH_3)$	C_1	3	3	1
	**90**	$O(SiHF_2)(SiH_3)$	C_s	3	3	1
	**91**	$O(SiHMePh)(SiH_3)$	C_1	18	18	1
	**92**	$O(SiH_2Me)_2$	C_{2v}	18	9	2
	**93**	$O(SiHMe_2)(SiH_2Me)$	C_1	27	27	1
	**94**	$O(SiH_2Ph)(SiH_2Me)$	C_s	6	6	1
	**95**	$O(SiMe_3)(SiH_2Me)$	C_s	243	243	1
	**96**	$O(SiHMe_2)_2$	C_2	162	81	2
	**97**	$O(SiMe_3)(SiHMe_2)$	C_1	729	729	1
	**98**	$O(SiMe_3)_2$	C_2	13122	6561	2
	**99**	$O(SiH_2Vi)_2$	C_1	1	1	1
	**100**	$O(SiH_2F)_2$	C_2	2	1	2
	**101**	$O(SiHF_2)(SiH_2F)$	C_1	1	1	1
	**102**	$O(SiF_3)(SiH_2F)$	C_1	3	3	1
	**103**	$O(SiHF_2)_2$	C_2	2	1	2
	**104**	$O(SiF_3)(SiHF_2)$	C_1	3	3	1
	**105**	$O(SiF_3)_2$	C_s	9	9	1
**XI**	**106**	$SiH_2(OSiH_3)_2$	C_2	18	9	2
	**107**	$SiH_2(OSiH_2Me)(OSiH_3)$	C_1	9	9	1
	**108**	$SiH_2(OSiH_2Vi)(OSiH_3)$	C_1	3	3	1
	**109**	$SiH_2(OSiH_2Ph)(OSiH_3)$	C_1	6	6	1
	**110**	$SiH_2(OSiH_2F)(OSiH_3)$	C_1	3	3	1
	**111**	$SiH_2(OSiMe_3)(OSiH_3)$	C_1	243	243	1
	**112**	$SiH_2(OSiHMe_2)(OSiH_3)$	C_1	27	27	1
	**113**	$SiH_2(OSiHF_2)(OSiH_3)$	C_1	3	3	1
	**114**	$SiH_2(OSiF_3)(OSiH_3)$	C_1	9	9	1
	**115**	$SiH_2(OSiH_2Me)_2$	C_2	18	9	2
	**116**	$SiH_2(OSiHMe_2)(OSiH_2Me)$	C_1	27	27	1
	**117**	$SiH_2(OSiMe_3)(OSiH_2Me)$	C_1	243	243	1

Table S2. Point groups and total, internal, and external symmetry numbers (σ_{tot}, σ_{int}, and σ_{ext}) of compounds **1**–**159**.

Group	#	Chemical Formula	Point Group	σ_{tot}	σ_{int}	σ_{ext}
	**118**	$SiH_2(OSiH_2F)_2$	C_2	2	1	2
	**119**	$SiH_2(OSiHMe_2)_2$	C_2	162	81	2
	**120**	$SiH_2(OSiMe_3)(OSiHMe_2)$	C_1	729	729	1
	**121**	$SiH_2(OSiMe_3)_2$	C_2	13122	6561	2
	**122**	$SiHMe(OSiH_3)_2$	C_1	27	27	1
	**123**	$SiHVi(OSiH_3)_2$	C_1	9	9	1
	**124**	$SiHPh(OSiH_3)_2$	C_1	18	18	1
	**125**	$SiHF(OSiH_3)_2$	C_1	9	9	1
	**126**	$SiHMe(OSiH_2Me)(OSiH_3)$	C_1	27	27	1
	**127**	$SiHMe(OSiHMe_2)(OSiH_3)$	C_1	81	81	1
	**128**	$SiHMe(OSiMe_3)(OSiH_3)$	C_1	729	729	1
	**129**	$SiHMe(OSiH_2Me)_2$	C_1	27	27	1
	**130**	$SiHMe(OSiHMe_2)(OSiH_2Me)$	C_1	81	81	1
	**131**	$SiHMe(OSiMe_3)(OSiH_2Me)$	C_1	729	729	1
	**132**	$SiHMe(OSiHMe_2)_2$	C_1	243	243	1
	**133**	$SiHMe(OSiMe_3)(OSiHMe_2)$	C_1	2187	2187	1
	**134**	$SiHMe(OSiMe_3)_2$	C_1	19683	19683	1
	**135**	$SiHF(OSiH_2F)(OSiH_3)$	C_1	3	3	1
	**136**	$SiHF(OSiHF_2)(OSiH_3)$	C_1	3	3	1
	**137**	$SiMe_2(OSiH_3)_2$	C_2	162	81	2
	**138**	$SiMe_2(OSiH_2Me)(OSiH_3)$	C_1	81	81	1
	**139**	$SiMe_2(OSiHMe_2)(OSiH_3)$	C_1	243	243	1
	**140**	$SiMe_2(OSiMe_3)(OSiH_3)$	C_1	2187	2187	1
	**141**	$SiMe_2(OSiH_2Me)_2$	C_2	162	81	2
	**142**	$SiMe_2(OSiHMe_2)(OSiH_2Me)$	C_1	243	243	1
	**143**	$SiMe_2(OSiMe_3)(OSiH_2Me)$	C_1	2187	2187	1
	**144**	$SiMe_2(OSiMe_3)_2$	C_1	59049	59049	1
	**145**	$SiMe_2(OSiHMe_2)_2$	C_1	729	729	1
	**146**	$SiMe_2(OSiMe_3)(OSiHMe_2)$	C_1	6561	6561	1
	**147**	$SiF_2(OSiH_3)_2$	C_{2v}	18	9	2
	**148**	$SiF_2(OSiH_2F)(OSiH_3)$	C_1	3	3	1
	**149**	$SiF_2(OSiH_2F)_2$	C_2	2	1	2
**XII**	**150**	$O(SiH_2OSiH_3)_2$	C_2	18	9	2
**XIII**	**151**	$(OSiH_2)_3$	D_{3h}	6	1	6
	**152**	$(OSiHMe)(OSiH_2)_2$	C_s	3	3	1
	**153**	$(OSiMe_2)(OSiH_2)_2$	C_{2v}	18	9	2
	**154**	$(OSiHMe)_2(OSiH_2)$	C_2	18	9	2
	**155**	$(OSiMe_2)(OSiHMe)(OSiH_2)$	C_1	27	27	1
	**156**	$(OSiHMe)_3$	C_s	27	27	1

Table S2. Point groups and total, internal, and external symmetry numbers (σ_{tot}, σ_{int}, and σ_{ext}) of compounds **1**–**159**.

Group	#	Chemical Formula	Point Group	σ_{tot}	σ_{int}	σ_{ext}
	**157**	$(OSiMe_2)_3$	D_{3h}	4374	729	6
**XIV**	**158**	$(OSiH_2)_4$	D_{2d}	4	1	4
**XV**	**159**	$NH(SiMe_3)_2$	C_2	13122	6561	2

Table S3. Calculated total energies (E_{tot}, 0 K, a.u.), zero-point vibrational energies (ZPE, a.u.), absolute enthalpies (H, 298 K, a.u.), thermal corrections to enthalpies (ΔH, 0–298 K, a.u.), and gas phase standard enthalpies of formation ($\Delta_f H°$, 298 K, kJ mol^{-1}) of compounds **1**–**159**.

			CBS-QB3		W1X-1			
**Group**	**#**	**Chemical Formula**	***H* 298 K**	**$\Delta_f H°$ 298 K**	**E_{tot} 0 K**	**ZPE**	**Δ*H* 0–298 K**	**$\Delta_f H°$ 298 K**
**I**	**1**	SiH_4	-291.413714	27.0	-292.309854	0.030668	0.034688	35.9
	**2**	SiH_3Me	-330.651616	-27.6	-331.644921	0.059923	0.065094	-23.8
	**3**	SiH_3Et	-369.871303	-34.4	-370.959916	0.088313	0.094717	-32.8
	**4**	SiH_3Vi	-368.661319	94.3	-369.726096	0.065754	0.071581	96.9
	**5**	SiH_3Ph	-522.032924	130.6	-523.408208	0.113389	0.121444	124.8
	**6**	$SiH_3{}^iPr$	-409.095376	-52.7	-410.278455	0.116000	0.123715	-52.7
	**7**	$SiH_3{}^sBu$	-448.31920	-70.2	-449.597202	0.144006	0.153083	-72.3
	**8**	SiH_3(3-Pe)	-487.542526	-86.8	-488.915534	0.172009	0.182501	-90.6
	**9**	SiH_2Me_2	-369.890599	-85.1	-370.980633	0.088504	0.095217	-85.9
	**10**	SiH_2EtMe	-409.110308	-91.9	-410.295620	0.116811	0.124871	-94.8
	**11**	SiH_2MeVi	-407.900633	35.9	-409.062171	0.094330	0.101769	34.0
	**12**	SiH_2MePh	-561.272920	70.5	-562.744771	0.142004	0.151647	63.3
	**13**	$SiH_2Me{}^iPr$	-448.334527	-110.7	-449.614227	0.144492	0.153885	-114.9
	**14**	$SiH_2Me{}^sBu$	-487.558217	-128.0	-488.932921	0.172506	0.18328	-134.2
	**15**	SiH_2Me(3-Pe)	-526.781346	-143.8	-528.250915	0.200535	0.212680	-151.7
	**16**	SiH_2Et_2	-448.330029	-98.8	-449.610586	0.145080	0.154515	-103.7
	**17**	SiH_2EtPh	-600.492896	62.9	-602.060044	0.170244	0.181308	52.7
	**18**	SiH_2Vi_2	-445.910788	156.6	-447.143908	0.100202	0.108355	153.5
	**19**	SiH_2Ph_2	-752.656240	223.4	-754.509791	0.195500	0.208112	210.3
	**20**	$SiHMe_3$	-409.130577	-145.2	-410.316878	0.116660	0.12513	-149.9
	**21**	$SiHEtMe_2$	-448.350330	-152.1	-449.631845	0.144967	0.154807	-158.7
	**22**	$SiHMe_2Vi$	-447.140853	-24.8	-448.398702	0.122489	0.131727	-30.6
	**23**	$SiHMe_2Ph$	-600.513596	8.5	-602.081664	0.170171	0.181649	-2.2
	**24**	$SiHMe_2{}^iPr$	-487.574606	-171.0	-488.950457	0.172668	0.183865	-178.7
	**25**	$SiHMe_2{}^sBu$	-526.798309	-188.4	-528.269090	0.200668	0.213257	-197.9
	**26**	$SiHMe_2$(3-Pe)	-566.021481	-204.3	-567.587073	0.228715	0.242678	-215.3
	**27**	$SiHEtMePh$	-639.734252	-0.8	-641.397289	0.198473	0.211311	-12.7
	**28**	$SiHMeVi_2$	-485.151290	95.1	-486.480715	0.128393	0.138362	88.2
	**29**	$SiHMePhVi$	-638.524535	127.2	-640.164016	0.176069	0.188266	115.7
	**30**	$SiHVi_3$	-523.161587	215.4	-524.562409	0.134145	0.14497	207.8
	**31**	$SiHPhVi_2$	-676.534818	247.5	-678.245847	0.181831	0.194825	234.9
	**32**	$SiMe_4$	-448.371377	-207.4	-449.653468	0.144668	0.15498	-215.0
	**33**	$SiEtMe_3$	-487.591122	-214.4	-488.968377	0.172893	0.184626	-223.7
	**34**	$SiMe_3Vi$	-486.381859	-87.6	-487.735549	0.150448	0.161563	-96.5
	**35**	$SiMe_3Ph$	-639.754963	-55.2	-641.418665	0.198146	0.211473	-68.4
	**36**	$SiMe_2Vi_2$	-524.392512	31.8	-525.817771	0.156328	0.168213	21.9
	**37**	$SiEtMe_2Ph$	-678.975746	-64.9	-680.734275	0.226486	0.241193	-78.8

Table S3. Calculated total energies (E_{tot}, 0 K, a.u.), zero-point vibrational energies (ZPE, a.u.), absolute enthalpies (H, 298 K, a.u.), thermal corrections to enthalpies (ΔH, 0–298 K, a.u.), and gas phase standard enthalpies of formation ($\Delta_f H°$, 298 K, kJ mol^{-1}) of compounds **1**–**159**.

			CBS-QB3		W1X-1			
Group	#	Chemical Formula	H 298 K	$\Delta_f H°$ 298 K	E_{tot} 0 K	ZPE	ΔH 0–298 K	$\Delta_f H°$ 298 K
	**38**	$SiMe_2PhVi$	-677.765986	63.2	-679.501198	0.204005	0.218133	49.1
	**39**	$SiMe_2Ph_2$	-831.139700	94.0	-833.184764	0.251645	0.268032	76.0
	**40**	$SiMeVi_3$	-562.403568	150.1	-563.900135	0.162217	0.17487	139.9
	**41**	$SiMePhVi_2$	-715.777480	180.4	-717.583935	0.209834	0.224763	166.1
	**42**	$SiEt_4$	-605.251715	-238.8	-606.914105	0.257956	0.273793	-251.9
**II**	**43**	Si_2H_6	-581.658769	74.2	-583.439624	0.048123	0.054156	81.1
	**44**	Si_2H_5Me	-620.896490	20.1	-622.774107	0.076895	0.084515	22.9
	**45**	$Si_2H_4Me_2$	-660.133953	-33.4	-662.108354	0.105644	0.114904	-34.6
	**46**	Si_2Me_6	-817.091408	-267.3	-819.449697	0.218209	0.234927	-280.3
**III**	**47**	Si_3H_8	-871.906768	113.7	-874.571872	0.065268	0.073808	120.4
**IV**	**48**	Si_4H_{10}	-1162.155299	151.7	-1165.704615	0.082352	0.093467	158.4
**V**	**49**	Si_5H_{12}	-1452.403983	189.4	-1456.837436	0.099402	0.113137	196.1
**VI**	**50**	SiH_3OH	-366.613728	-286.7	-367.636710	0.037857	0.042664	-280.1
	**51**	SiH_2MeOH	-405.855845	-352.4	-406.975551	0.066338	0.072659	-350.7
	**52**	SiH_2EtOH	-445.075586	-359.3	-446.290427	0.094734	0.102341	-359.3
	**53**	$SiHMe_2OH$	-445.097827	-417.7	-446.313591	0.094399	0.102478	-419.7
	**54**	$SiMe_3OH$	-484.339943	-483.4	-485.651344	0.122253	0.132218	-488.2
	**55**	SiH_3OMe	-405.818260	-253.7	-406.935060	0.065876	0.072149	-245.8
	**56**	$SiH_2Me(OMe)$	-445.060494	-319.7	-446.273900	0.094254	0.102154	-316.4
	**57**	$SiHMe_2(OMe)$	-484.302416	-384.9	-485.611826	0.122296	0.131989	-385.0
**VII**	**58**	$SiH_2(OH)_2$	-441.826234	-633.2	-442.975863	0.044881	0.050517	-628.7
	**59**	$SiH_2(OMe)_2$	-520.234722	-565.7	-521.571732	0.100532	0.109519	-557.7
	**60**	$SiHMe(OMe)_2$	-559.478226	-635.1	-560.911047	0.128387	0.13926	-630.3
	**61**	$SiHVi(OMe)_2$	-597.488065	-513.6	-598.992414	0.134392	0.145988	-509.5
	**62**	$SiHPh(OMe)_2$	-750.861769	-482.7	-752.676231	0.182063	0.195891	-483.3
	**63**	$SiMe_2(OMe)_2$	-598.721994	-705.1	-600.250069	0.156119	0.168918	-702.3
	**64**	$SiMeVi(OMe)_2$	-636.732212	-584.6	-638.331758	0.162079	0.175636	-582.4
	**65**	$SiMePh(OMe)_2$	-790.106387	-555.0	-792.015793	0.208062	0.225525	-556.8
	**66**	$SiVi_2(OMe)_2$	-674.742516	-464.3	-676.413521	0.168031	0.182361	-462.6
	**67**	$SiPhVi(OMe)_2$	-828.116947	-435.4	-830.097701	0.215694	0.232264	-437.4
	**68**	$SiPh_2(OMe)_2$	-981.491560	-406.9	-983.781967	0.263195	0.282132	-412.4
**VIII**	**69**	$SiH(OH)_3$	-517.042175	-988.7	-518.3176724	0.050716	0.057712	-985.9
	**70**	$SiMe(OMe)_2OH$	-634.694826	-992.3	-636.252516	0.134138	0.146487	-986.6
	**71**	$SiEt(OMe)_2OH$	-673.914525	-999.1	-675.567158	0.162499	0.17624	-994.3
	**72**	$SiMe(OMe)_3$	-673.898455	-957.0	-675.549655	0.162178	0.176149	-948.6
	**73**	$SiEt(OMe)_3$	-713.118347	-964.3	-714.864408	0.190509	0.205885	-956.7
**IX**	**74**	$Si(OH)_4$	-592.258099	-1344.2	-593.659022	0.056862	0.065022	-1341.7

Table S3. Calculated total energies (E_{tot}, 0 K, a.u.), zero-point vibrational energies (ZPE, a.u.), absolute enthalpies (H, 298 K, a.u.), thermal corrections to enthalpies (ΔH, 0–298 K, a.u.), and gas phase standard enthalpies of formation ($\Delta_f H°$, 298 K, kJ mol^{-1}) of compounds **1**–**159**.

			CBS-QB3		W1X-1			
**Group**	**#**	**Chemical Formula**	***H*** **298 K**	**$\Delta_f H°$ 298 K**	**E_{tot} 0 K**	**ZPE**	**ΔH 0–298 K**	**$\Delta_f H°$ 298 K**
	**75**	$Si(OMe)_3OH$	-709.870983	-1243.4	-711.551950	0.140504	0.153806	-1232.3
	**76**	$Si(OEt)(OMe)_2OH$	-749.100881	-1277.0	-750.876416	0.168367	0.182914	-1267.5
	**77**	$Si(OEt)_2(OMe)OH$	-788.330838	-1310.8	-790.200928	0.196267	0.212037	-1302.8
	**78**	$Si(OMe)_4$	-749.075316	-1209.9	-750.849625	0.168414	0.183432	-1195.9
	**79**	$Si(OEt)(OMe)_3$	-788.305318	-1243.8	-790.174168	0.196289	0.212534	-1231.3
	**80**	$Si(OEt)_4$	-905.995438	-1345.7	-908.147859	0.279911	0.299868	-1337.7
**X**	**81**	$O(SiH_3)_2$	-656.903269	-356.3	-658.805356	0.053868	0.061070	-339.7
	**82**	$O(SiMe_3)(SiH_3)$	-774.630936	-556.8	-776.821064	0.138142	0.150598	-550.7
	**83**	$O(SiF_3)(SiH_3)$	-954.665810	-1620.9	-957.030648	0.039124	0.048322	-1605.9
	**84**	$O(SiH_2Me)(SiH_3)$	-696.145524	-422.3	-698.144321	0.082330	0.091035	-410.7
	**85**	$O(SiH_2Vi)(SiH_3)$	-734.155073	-300.1	-736.225305	0.088233	0.097618	-289.3
	**86**	$O(SiH_2Ph)(SiH_3)$	-887.527461	-265.8	-889.907884	0.135940	0.147596	-259.6
	**87**	$O(SiH_2F)(SiH_3)$	-756.153777	-774.9	-758.213389	0.049575	0.057187	-759.8
	**88**	$O(SiHMe_2)(SiH_3)$	-735.388131	-489.3	-737.482858	0.110333	0.120859	-481.0
	**89**	$O(SiHVi_2)(SiH_3)$	-811.408462	-248.0	-813.646179	0.122255	0.134177	-241.3
	**90**	$O(SiHF_2)(SiH_3)$	-855.408291	-1204.1	-857.625032	0.044628	0.052931	-1190.3
	**91**	$O(SiHMePh)(SiH_3)$	-926.771182	-335.7	-929.247445	0.163919	0.177403	-332.7
	**92**	$O(SiH_2Me)_2$	-735.387733	-488.3	-737.483157	0.110740	0.121016	-481.4
	**93**	$O(SiHMe_2)(SiH_2Me)$	-774.630275	-555.1	-776.821295	0.138753	0.150829	-550.7
	**94**	$O(SiH_2Ph)(SiH_2Me)$	-926.769249	-330.6	-929.246535	0.164240	0.177536	-329.9
	**95**	$O(SiMe_3)(SiH_2Me)$	-813.873029	-622.5	-816.159847	0.166567	0.180566	-621.2
	**96**	$O(SiHMe_2)_2$	-813.872720	-621.7	-816.160084	0.166742	0.180639	-621.6
	**97**	$O(SiMe_3)(SiHMe_2)$	-853.115501	-689.1	-855.498102	0.194523	0.210375	-690.8
	**98**	$O(SiMe_3)_2$	-892.358162	-756.2	-894.836113	0.222347	0.240110	-760.0
	**99**	$O(SiH_2Vi)_2$	-811.407110	-244.5	-813.645357	0.122582	0.134259	-238.9
	**100**	$O(SiH_2F)_2$	-855.403719	-1192.1	-857.621117	0.045208	0.053281	-1179.1
	**101**	$O(SiHF_2)(SiH_2F)$	-954.657656	-1619.7	-957.031961	0.040136	0.048975	-1607.7
	**102**	$O(SiF_3)(SiH_2F)$	-1053.907041	-2035.4	-1056.437208	0.034744	0.044358	-2022.4
	**103**	$O(SiHF_2)_2$	-1053.910908	-2045.5	-1056.442186	0.035180	0.044672	-2034.6
	**104**	$O(SiF_3)(SiHF_2)$	-1153.160091	-2460.7	-1155.847232	0.029752	0.040040	-2448.8
	**105**	$O(SiF_3)_2$	-1252.408639	-2874.1	-1255.251658	0.024249	0.035410	-2861.4
**XI**	**106**	$SiH_2(OSiH_3)_2$	-1022.405034	-771.7	-1025.312308	0.076537	0.087137	-746.1
	**107**	$SiH_2(OSiH_2Me)(OSiH_3)$	-1061.647384	-838.0	-1064.650971	0.104933	0.117113	-816.3
	**108**	$SiH_2(OSiH_2Vi)(OSiH_3)$	-1099.657041	-716.0	-1102.732341	0.110803	0.123688	-695.9
	**109**	$SiH_2(OSiH_2Ph)(OSiH_3)$	-1253.028903	-680.3	-1256.414654	0.158452	0.173642	-665.7
	**110**	$SiH_2(OSiH_2F)(OSiH_3)$	-1121.655751	-1190.8	-1124.720245	0.072090	0.083239	-1166.0
	**111**	$SiH_2(OSiMe_3)(OSiH_3)$	-1140.133274	-973.7	-1143.328541	0.160765	0.176674	-958.5

Table S3. Calculated total energies (E_{tot}, 0 K, a.u.), zero-point vibrational energies (ZPE, a.u.), absolute enthalpies (H, 298 K, a.u.), thermal corrections to enthalpies (ΔH, 0–298 K, a.u.), and gas phase standard enthalpies of formation ($\Delta_f H°$, 298 K, kJ mol^{-1}) of compounds **1**–**159**.

			CBS-QB3		W1X-1			
Group	#	Chemical Formula	H 298 K	$\Delta_f H°$ 298 K	E_{tot} 0 K	ZPE	ΔH 0–298 K	$\Delta_f H°$ 298 K
	**112**	$SiH_2(OSiHMe_2)(OSiH_3)$	-1100.890297	-905.8	-1103.990219	0.132942	0.146930	-888.5
	**113**	$SiH_2(OSiHF_2)(OSiH_3)$	-1220.910302	-1620.1	-1224.131718	0.067171	0.078980	-1596.1
	**114**	$SiH_2(OSiF_3)(OSiH_3)$	-1320.160038	-2036.7	-1323.537374	0.061716	0.074356	-2011.9
	**115**	$SiH_2(OSiH_2Me)_2$	-1100.889691	-904.2	-1103.990257	0.133331	0.147090	-888.2
	**116**	$SiH_2(OSiHMe_2)(OSiH_2Me)$	-1140.132604	-972.0	-1143.329181	0.161338	0.176906	-959.5
	**117**	$SiH_2(OSiMe_3)(OSiH_2Me)$	-1179.375570	-1039.9	-1182.667463	0.189136	0.206645	-1029.4
	**118**	$SiH_2(OSiH_2F)_2$	-1220.905379	-1607.1	-1224.127897	0.067703	0.079361	-1585.0
	**119**	$SiH_2(OSiHMe_2)_2$	-1179.375376	-1039.4	-1182.667897	0.189321	0.206714	-1030.3
	**120**	$SiH_2(OSiMe_3)(OSiHMe_2)$	-1218.618310	-1107.2	-1222.006176	0.217139	0.236457	-1100.2
	**121**	$SiH_2(OSiMe_3)_2$	-1257.861270	-1175.1	-1261.344416	0.244951	0.266201	-1169.9
	**122**	$SiHMe(OSiH_3)_2$	-1061.649371	-843.2	-1064.652496	0.104266	0.116815	-821.1
	**123**	$SiHVi(OSiH_3)_2$	-1099.658885	-720.8	-1102.733354	0.110153	0.123459	-699.2
	**124**	$SiHPh(OSiH_3)_2$	-1253.032214	-689.0	-1256.416751	0.157891	0.173368	-671.9
	**125**	$SiHF(OSiH_3)_2$	-1121.660607	-1203.6	-1124.724608	0.071477	0.082921	-1178.3
	**126**	$SiHMe(OSiH_2Me)(OSiH_3)$	-1100.891737	-909.5	-1103.991455	0.132696	0.146792	-892.1
	**127**	$SiHMe(OSiHMe_2)(OSiH_3)$	-1140.134534	-977.0	-1143.330363	0.160761	0.176612	-963.4
	**128**	$SiHMe(OSiMe_3)(OSiH_3)$	-1179.377576	-1045.1	-1182.668558	0.188491	0.206341	-1033.0
	**129**	$SiHMe(OSiH_2Me)_2$	-1140.133969	-975.5	-1143.330305	0.161083	0.176763	-962.9
	**130**	$SiHMe(OSiHMe_2)(OSiH_2Me)$	-1179.376922	-1043.4	-1182.669305	0.189156	0.206591	-1034.4
	**131**	$SiHMe(OSiMe_3)(OSiH_2Me)$	-1218.619879	-1111.3	-1222.007526	0.216924	0.236321	-1104.1
	**132**	$SiHMe(OSiHMe_2)_2$	-1218.619614	-1110.6	-1222.007756	0.217165	0.236411	-1104.4
	**133**	$SiHMe(OSiMe_3)(OSiHMe_2)$	-1257.862788	-1179.1	-1261.346183	0.244974	0.266152	-1174.7
	**134**	$SiHMe(OSiMe_3)_2$	-1297.105549	-1246.5	-1300.684160	0.272674	0.295858	-1243.8
	**135**	$SiHF(OSiH_2F)(OSiH_3)$	-1220.911365	-1622.9	-1224.131256	0.067209	0.079008	-1594.8
	**136**	$SiHF(OSiHF_2)(OSiH_3)$	-1320.165526	-2051.1	-1323.543527	0.062127	0.074702	-2027.1
	**137**	$SiMe_2(OSiH_3)_2$	-1100.894003	-915.5	-1103.992140	0.132081	0.146484	-894.7
	**138**	$SiMe_2(OSiH_2Me)(OSiH_3)$	-1140.136222	-981.5	-1143.330991	0.160465	0.176454	-965.5
	**139**	$SiMe_2(OSiHMe_2)(OSiH_3)$	-1179.379069	-1049.1	-1182.669840	0.188522	0.206278	-1036.6
	**140**	$SiMe_2(OSiMe_3)(OSiH_3)$	-1218.622011	-1116.9	-1222.008038	0.216316	0.236016	-1106.2
	**141**	$SiMe_2(OSiH_2Me)_2$	-1179.378418	-1047.4	-1182.669757	0.188860	0.206420	-1036.0
	**142**	$SiMe_2(OSiHMe_2)(OSiH_2Me)$	-1218.621294	-1115.0	-1222.008651	0.216911	0.236251	-1107.2
	**143**	$SiMe_2(OSiMe_3)(OSiH_2Me)$	-1257.864207	-1182.8	-1261.346811	0.244690	0.265984	-1176.7
	**144**	$SiMe_2(OSiMe_3)_2$	-1336.349853	-1317.9	-1340.023476	0.300459	0.325513	-1316.6
	**145**	$SiMe_2(OSiHMe_2)_2$	-1257.863984	-1182.3	-1261.347041	0.244862	0.266061	-1177.1
	**146**	$SiMe_2(OSiMe_3)(OSiHMe_2)$	-1297.107042	-1250.4	-1300.685272	0.272651	0.295795	-1246.9
	**147**	$SiF_2(OSiH_3)_2$	-1220.912338	-1625.4	-1224.132134	0.066042	0.078336	-1598.9
	**148**	$SiF_2(OSiH_2F)(OSiH_3)$	-1320.162749	-2043.8	-1323.539393	0.061638	0.074395	-2017.1

Table S3. Calculated total energies (E_{tot}, 0 K, a.u.), zero-point vibrational energies (ZPE, a.u.), absolute enthalpies (H, 298 K, a.u.), thermal corrections to enthalpies (ΔH, 0–298 K, a.u.), and gas phase standard enthalpies of formation ($\Delta_f H°$, 298 K, kJ mol^{-1}) of compounds **1**–**159**.

			CBS-QB3		W1X-1			
**Group**	**#**	**Chemical Formula**	***H* 298 K**	**$\Delta_f H°$ 298 K**	**E_{tot} 0 K**	**ZPE**	**ΔH 0–298 K**	**$\Delta_f H°$ 298 K**
	**149**	$SiF_2(OSiH_2F)_2$	-1419.412533	-2460.5	-1422.946504	0.057191	0.070437	-2434.9
**XII**	**150**	$O(SiH_2OSiH_3)_2$	-1387.906454	-1186.1	-1391.818993	0.099035	0.113186	-1151.9
**XIII**	**151**	$(OSiH_2)_3$	-1096.493698	-1215.7	-1099.509972	0.067501	0.076059	-1196.4
	**152**	$(OSiHMe)(OSiH_2)_2$	-1135.739120	-1290.0	-1138.850990	0.095348	0.105780	-1273.4
	**153**	$(OSiMe_2)(OSiH_2)_2$	-1174.983966	-1362.9	-1178.191047	0.123070	0.135456	-1348.1
	**154**	$(OSiHMe)_2(OSiH_2)$	-1174.984316	-1363.8	-1178.191887	0.123173	0.135493	-1350.2
	**155**	$(OSiMe_2)(OSiHMe)(OSiH_2)$	-1214.229128	-1436.6	-1217.531818	0.150898	0.165170	-1424.6
	**156**	$(OSiHMe)_3$	-1214.229392	-1437.3	-1217.532644	0.151009	0.165212	-1426.6
	**157**	$(OSiMe_2)_3$	-1331.96313	-1653.8	-1335.551897	0.234084	0.254204	-1648.3
**XIV**	**158**	$(OSiH_2)_4$	-1462.005044	-1656.2	-1466.024979	0.089813	0.102309	-1623.5
**XV**	**159**	$NH(SiMe_3)_2$	-872.441223	-454.0	-874.901159	0.233771	0.251706	-472.0

Table S4. Comparison between experimental (Exptl.) and estimated (Benson) standard gas phase enthalpies of formation ($\Delta_f H°$, 298 K, kJ mol^{-1}) of organosilicon compounds studied by Voronkov *et al.*[a]

Chemical Formula	Benson Groups[b, c]	$\Delta_f H°$ 298 K Exptl.	$\Delta_f H°$ 298 K Benson	Diff.
$SiH(C_4H_9)_3$	6*C-(C)$_2$(H)$_2$, 3*C-(C)(H)$_3$, 3*C-(C)(H)$_2$(Si), Si-(H)(C)$_3$	-341.0	-301	40
$SiH(C_5H_{11})_3$	9*C-(C)$_2$(H)$_2$, 3*C-(C)(H)$_3$, 3*C-(C)(H)$_2$(Si), Si-(H)(C)$_3$	-402.0	-362	40
$SiH(C_6H_{13})_3$	12*C-(C)$_2$(H)$_2$, 3*C-(C)(H)$_3$, 3*C-(C)(H)$_2$(Si), Si-(H)(C)$_3$	-466.0	-424	42
$SiH(C_7H_{15})_3$	15*C-(C)$_2$(H)$_2$, 3*C-(C)(H)$_3$, 3*C-(C)(H)$_2$(Si), Si-(H)(C)$_3$	-529.0	-486	43
$SiH(C_8H_{17})_3$	18*C-(C)$_2$(H)$_2$, 3*C-(C)(H)$_3$, 3*C-(C)(H)$_2$(Si), Si-(H)(C)$_3$	-591.0	-548	43
$SiH(C_9H_{19})_3$	21*C-(C)$_2$(H)$_2$, 3*C-(C)(H)$_3$, 3*C-(C)(H)$_2$(Si), Si-(H)(C)$_3$	-651.0	-610	41
$SiH(C_{10}H_{21})_3$	24*C-(C)$_2$(H)$_2$, 3*C-(C)(H)$_3$, 3*C-(C)(H)$_2$(Si), Si-(H)(C)$_3$	-713.0	-672	41
SiH(*s*-C_4H_9)$_3$	6*C-(C)(H)$_3$, 3*C-(C)$_3$(H), 3*tert., 3*C-(C)(H)$_2$(Si), Si-(H)(C)$_3$	-355.0	-314	41
SiH(*i*-C_5H_{11})$_3$	6*C-(C)(H)$_3$, 3*C-(C)$_2$(H)$_2$, 3*C-(C)$_3$(H), 3*tert., 3*C-(C)(H)$_2$(Si), Si-(H)(C)$_3$	-413.0	-376	37
$SiH(CH_3)(C_4H_9)_2$	4*C-(C)$_2$(H)$_2$, 2*C-(C)(H)$_3$, C-(H)$_3$(Si), 2*C-(C)(H)$_2$(Si), Si-(H)(C)$_3$	-283.0	-250	33
$SiH(CH_3)(C_5H_{11})_2$	6*C-(C)$_2$(H)$_2$, 2*C-(C)(H)$_3$, C-(H)$_3$(Si), 2*C-(C)(H)$_2$(Si), Si-(H)(C)$_3$	-325.0	-292	33
$SiH(CH_3)(C_6H_{13})_2$	8*C-(C)$_2$(H)$_2$, 2*C-(C)(H)$_3$, C-(H)$_3$(Si), 2*C-(C)(H)$_2$(Si), Si-(H)(C)$_3$	-366.0	-333	33
$SiH(CH_3)(C_{10}H_{21})_2$	16*C-(C)$_2$(H)$_2$, 2*C-(C)(H)$_3$, C-(H)$_3$(Si), 2*C-(C)(H)$_2$(Si), Si-(H)(C)$_3$	-531.0	-498	33
$SiH(C_2H_5)(C_4H_9)_2$	4*C-(C)$_2$(H)$_2$, 3*C-(C)(H)$_3$, 3*C-(C)(H)$_2$(Si), Si-(H)(C)$_3$	-301.0	-259	42
$SiH(C_2H_5)(C_5H_{11})_2$	6*C-(C)$_2$(H)$_2$, 3*C-(C)(H)$_3$, 3*C-(C)(H)$_2$(Si), Si-(H)(C)$_3$	-340.0	-301	39
$SiH(C_2H_5)(C_6H_{13})_2$	8*C-(C)$_2$(H)$_2$, 3*C-(C)(H)$_3$, 3*C-(C)(H)$_2$(Si), Si-(H)(C)$_3$	-381.0	-342	39
$SiH(C_2H_5)(C_8H_{17})_2$	12*C-(C)$_2$(H)$_2$, 3*C-(C)(H)$_3$, 3*C-(C)(H)$_2$(Si), Si-(H)(C)$_3$	-468.0	-424	44
$SiH(C_2H_5)(C_{10}H_{21})_2$	16*C-(C)$_2$(H)$_2$, 3*C-(C)(H)$_3$, 3*C-(C)(H)$_2$(Si), Si-(H)(C)$_3$	-545.0	-507	38
$SiH(C_2H_5)$(*s*-C_4H_9)$_2$	5*C-(C)(H)$_3$, 2*C-(C)$_3$(H), 2*tert., 3*C-(C)(H)$_2$(Si), Si-(H)(C)$_3$	-315.0	-268	47
$SiH(C_2H_5)$(*i*-C_5H_{11})$_2$	5*C-(C)(H)$_3$, 2*C-(C)$_2$(H)$_2$, 2*C-(C)$_3$(H), 2*tert., 3*C-(C)(H)$_2$(Si), Si-(H)(C)$_3$	-358.0	-309	49
$Si(C_3H_7)_2(C_4H_9)_2$	6*C-(C)$_2$(H)$_2$, 4*C-(C)(H)$_3$, 4*C-(C)(H)$_2$(Si), Si-(C)$_4$	-423.0	-375	48
$Si(C_3H_7)(C_4H_9)_3$	7*C-(C)$_2$(H)$_2$, 4*C-(C)(H)$_3$, 4*C-(C)(H)$_2$(Si), Si-(C)$_4$	-444.0	-395	49
$Si(C_3H_7)_2(OC_2H_5)_2$	4*C-(C)(H)$_3$, 2*C-(C)$_2$(H)$_2$, 2*C-(C)(H)$_2$(O), 2*C-(C)(H)$_2$(Si), 2*O-(C)(Si), Si-(C)$_2$(O)$_2$	-852.0	-829	23
$Si(OC_3H_7)_4$	4*C-(C)(H)$_3$, 4*C-(C)$_2$(H)$_2$, 4*C-(C)(H)$_2$(O), 4*O-(C)(Si), Si-(O)$_4$	-1397.0	-1413	-16
$Si(OC_4H_9)_4$	8*C-(C)$_2$(H)$_2$, 4*C-(C)(H)$_3$, 4*C-(C)(H)$_2$(O), 4*O-(C)(Si), Si-(O)$_4$	-1482.0	-1496	-14
$(OSiPh_2)_3$	30*C$_B$-(C$_B$)$_2$(H), 3*[Si-(C$_B$)$_2$(O)$_2$ + C$_B$-(C$_B$)$_2$(Si)], 3*O-(Si)$_2$, 6-mbr.	-880.0	-771	109
$(OSiMe_2)_4$	8*C-(H)$_3$(Si), 4*Si-(C)$_2$(O)$_2$, 4*O-(Si)$_2$, 8-mbr.	-2138.0	-2218	-80
$(OSiMe_2)(OSiPh_2)_3$	30*C$_B$-(C$_B$)$_2$(H), 2*C-(H)$_3$(Si), 4*O-(Si)$_2$, 3*[Si-(C$_B$)$_2$(O)$_2$ + C$_B$-(C$_B$)$_2$(Si)], Si-(C)$_2$(O)$_2$, 8-mbr.	-1454.0	-1343	111
$(OSiMe_2)_2(OSiPh_2)_2$	20*C$_B$-(C$_B$)$_2$(H), 4*C-(H)$_3$(Si), 4*O-(Si)$_2$, 2*Si-(C)$_2$(O)$_2$, 2*[Si-(C$_B$)$_2$(O)$_2$ + C$_B$-(C$_B$)$_2$(Si)], 8-mbr.	-1691.0	-1635	56
$(OSiMe_2)_3(OSiPh_2)$	10*C$_B$-(C$_B$)$_2$(H), 6*C-(H)$_3$(Si), 4*O-(Si)$_2$, 3*Si-(C)$_2$(O)$_2$, [Si-(C$_B$)$_2$(O)$_2$ + C$_B$-(C$_B$)$_2$(Si)], 8-mbr.	-1910.0	-1926	-16
$(OSiPh_2)_4$	40*C$_B$-(C$_B$)$_2$(H), 4*[Si-(C$_B$)$_2$(O)$_2$ + C$_B$-(C$_B$)$_2$(Si)], 4*O-(Si)$_2$, 8-mbr.	-1226.0	-1052	174
$(OSiMePh)_4$	4*C-(H)$_3$(Si), 20*C$_B$-(C$_B$)$_2$(H), 4*[Si-(C$_B$)$_2$(O)$_2$ + C$_B$-(C$_B$)$_2$(Si)], 4*O-(Si)$_2$, 8-mbr.	-1685.0	-1629	56
$Si(OCH_3)_3[(CH_2)_2SCH_3]$	3*C-(H)$_3$(O), C-(C)(H)$_2$(S), C-(H)$_3$(S), S-(C)$_2$, 3*O-(C)(Si), C-(C)(H)$_2$(Si), Si-(C)(O)$_3$	-946.6	-933	13
$Si(OCH_3)_3[(CH_2)_3SCH_3]$	3*C-(H)$_3$(O), C-(C)$_2$(H)$_2$, C-(C)(H)$_2$(S), C-(H)$_3$(S), S-(C)$_2$, 3*O-(C)(Si), C-(C)(H)$_2$(Si), Si-(C)(O)$_3$	-957.0	-954	3
$Si(OCH_3)_3[(CH_2)_2S(CH_2CH_3)]$	3*C-(H)$_3$(O), 2*C-(C)(H)$_2$(S), C-(H)$_3$(C), S-(C)$_2$, 3*O-(C)(Si), C-(C)(H)$_2$(Si), Si-(C)(O)$_3$	-962.2	-956	6
$Si(OCH_3)_3[(CH_2)_3S(CH_2CH_3)]$	3*C-(H)$_3$(O), 2*C-(C)(H)$_2$(S), C-(C)$_2$(H)$_2$, C-(H)$_3$(C), S-(C)$_2$, 3*O-(C)(Si), C-(C)(H)$_2$(Si), Si-(C)(O)$_3$	-979.9	-977	3
$Si(OCH_2CH_3)_3[(CH_2)_2S(CH_2CH_3)]$	4*C-(C)(H)$_3$,3*C-(C)(H)$_2$(O), 2*C-(C)(H)$_2$(S), S-(C)$_2$, 3*O-(C)(Si), C-(C)(H)$_2$(Si), Si-(C)(O)$_3$	-1069.0	-1055	14

Table S4. Comparison between experimental (Exptl.) and estimated (Benson) standard gas phase enthalpies of formation ($\Delta_f H°$, 298 K, kJ mol^{-1}) of organosilicon compounds studied by Voronkov *et al.*[a]

Chemical Formula	Benson Groups[b, c]	$\Delta_f H°$ 298 K Exptl.	$\Delta_f H°$ 298 K Benson	Diff.
$Si(OCH_2CH_3)_3[(CH_2)_3S(CH_2CH_3)]$	4*C-(C)$(H)_3$, 3*C-$(C)_2(H)_2$, 3*C-(C)$(H)_2$(O), 2*C-(C)$(H)_2$(S), S-$(C)_2$, *3*O-(C)(Si), C-(C)$(H)_2$(Si), Si-(C)$(O)_3$*	-1119.0	-1117	2

[a] See references 20–25 for details of the experimental work.

[b] Literature values (kJ mol^{-1}): C-(C)$(H)_3$ = C-$(H)_3$(O) = C-$(H)_3$(Si) = −42.26, C-$(C)_4$ = 19.2, C-$(C)_3$(H) = −1.17, C-$(C)_2(H)_2$ = −20.63, C_B-$(C_B)_2$(H) = −13.81, C-(C)$(H)_2$(O) = −32.90, C-(C)$(H)_2$(S) = −23.17, S-$(C)_2$ = 46.99., tert. corr = −2.26.

[c] Determined in this work (*italicized*, kJ mol^{-1}): C-$(C)_2$(H)(Si) = 17, C-(C)$(H)_2$(Si) = −9, Si-$(C)_4$ = −46, Si-$(C)_3$(H) = −23, Si-$(C)_2(O)_2$ = −55, Si-(C)$(C_B)(O)_2$ = −19, [Si-$(C_B)_2(O)_2$ + C_B-$(C_B)_2$(Si)] = 14, Si-(C)$(O)_3$ = −59, Si-(C)(H)$(O)_2$ = −24, Si-$(O)_4$ = −70, O-(C)(Si) = −240, O-$(Si)_2$ = −416, 6-mbr. ring corr. = 21, 8-mbr. ring corr. = 4.
